# HOXD12 defines an age-related aggressive subtype of oligodendroglioma

**DOI:** 10.1007/s00401-024-02802-1

**Published:** 2024-09-11

**Authors:** Nicholas Nuechterlein, Sadie Cimino, Allison Shelbourn, Vinny Ha, Sonali Arora, Sharika Rajan, Linda G. Shapiro, Eric C. Holland, Kenneth Aldape, Tresa McGranahan, Mark R. Gilbert, Patrick J. Cimino

**Affiliations:** 1 Neuropathology Unit, Surgical Neurology Branch, National Institute of Neurological Disorders and Stroke, National Institutes of Health, 10 Center Drive, Building 10/3D17, Bethesda, MD 20892 USA; 2 School of Interdisciplinary Arts and Sciences, University of Washington, Bothell, WA USA; 3 Division of Human Biology, Fred Hutchinson Cancer Research Center, Seattle, WA USA; 4 Laboratory of Pathology, National Cancer Institute, National Institutes of Health, Bethesda, MD USA; 5 Paul G. Allen School of Computer Science and Engineering, University of Washington, Seattle, WA USA; 6 Division of Hematology and Oncology, Scripps Cancer Center, La Jolla, CA USA; 7 Neuro-Oncology Branch, National Cancer Institute, National Institutes of Health, Bethesda, MD USA

**Keywords:** Oligodendroglioma, HOXD12, HOX, RNA-Seq, DNA methylation, ATAC-seq

## Abstract

Oligodendroglioma, IDH-mutant and 1p/19q-codeleted has highly variable outcomes that are strongly influenced by patient age. The distribution of oligodendroglioma age is non-Gaussian and reportedly bimodal, which motivated our investigation of age-associated molecular alterations that may drive poorer outcomes. We found that elevated HOXD12 expression was associated with both older patient age and shorter survival in the TCGA (FDR < 0.01, FDR = 1e−5) and the CGGA (*p* = 0.03, *p* < 1e−3). *HOXD12* gene body hypermethylation was associated with older age, higher WHO grade, and shorter survival in the TCGA (*p* < 1e−6, *p* < 0.001, *p* < 1e−3) and with older age and higher WHO grade in Capper et al. (*p* < 0.002, *p* = 0.014). In the TCGA, *HOXD12* gene body hypermethylation and elevated expression were independently prognostic of *NOTCH1* and *PIK3CA* mutations, loss of 15q, MYC activation, and standard histopathological features. Single-nucleus RNA and ATAC sequencing data showed that HOXD12 activity was elevated in neoplastic tissue, particularly within cycling and OPC-like cells, and was associated with a stem-like phenotype. A pan-HOX DNA methylation analysis revealed an age and survival-associated HOX-high signature that was tightly associated with *HOXD12* gene body methylation. Overall, HOXD12 expression and gene body hypermethylation were associated with an older, atypically aggressive subtype of oligodendroglioma.

**Supplementary Information:**

The online version contains supplementary material available at 10.1007/s00401-024-02802-1.

## Introduction

Oligodendroglioma, IDH-mutant and 1p/19q-codeleted is a type of adult-type diffuse glioma molecularly distinct from IDH-mutant astrocytoma and IDH-wildtype glioblastoma [43]. Patients diagnosed with oligodendroglioma generally have favorable outcomes compared to other adult-type diffuse gliomas, but individual patient outcomes are highly variable, with some patients living less than a year and others living decades [2, 11, 23, 30]. Chemoradiation, considered the standard of care treatment for oligodendrogliomas, is associated with short-term and long-term functional and cognitive toxicities [89]. Considering the toxicity of this non-curative treatment and the relatively favorable prognosis of oligodendroglioma, current guidelines offer varying definitions of high-risk tumors, which results in situations where it is unclear whether a patient should receive postoperative chemoradiation or whether IDH inhibitors, chemotherapy, or observation are more appropriate [49, 51, 83, 89]. For example, the American Society for Radiation Oncology (ASTRO) recommends all patients over age 40 should be treated with chemoradiation [31]; however, several other guidelines support delaying chemoradiation until disease progression based on patient and provider preference [51, 52, 83]. Patient-specific risk indicators are needed to identify patients with abnormally aggressive disease for whom the delay of chemoradiation would not be appropriate.

Oligodendroglioma age has been reported to be prognostic and bimodally distributed [17]. Based on previous studies of IDH-wildtype glioblastoma, we hypothesized that older oligodendroglioma patients may have molecular signatures indicative of poor survival [16, 58]. To date, only a few prognostic molecular biomarkers have been proposed (including *PIK3CA* mutations [74], *NOTCH1* mutations [2, 30], *CDKN2A* homozygous deletion [3], loss of 15q [30], and *MYC* activation [38]) to complement known clinical factors associated with survival such as age, KPS, World Health Organization (WHO) grade, and extent of resection [17, 26]. In this study, we sought to discover and validate novel age-associated alterations that characterize aggressive oligodendroglioma. These age-related molecular features may reflect a more aggressive subtype of oligodendroglioma that occurs disproportionately in older patients. To interrogate this hypothesis, we investigated oligodendroglioma data across multiple cohorts, including those published by The Cancer Genome Atlas (TCGA), the Chinese Glioma Genome Atlas (CGGA), the Glioma Longitudinal AnalySiS consortium (GLASS), and Capper et al. along with single-nucleus sequencing data published by Blanco-Carmona et al. and Wang et al. to identify, validate, and characterize prognostically relevant genes [10, 13, 19, 82, 91].

## Materials and methods

### The cancer genome atlas (TCGA) molecular and clinical data

Precomputed DNA methylation beta values (Illumina 450 K) from 171 primary oligodendroglioma, IDH-mutant and 1p/19q-codeleted tumors from TCGA were downloaded from University of California Santa Cruz (UCSC) Xena (https://xena.ucsc.edu/) [28]. RNA-seq gene counts for TCGA oligodendroglioma were downloaded from the National Cancer Institute’s Genomic Data Commons (GDC) Data Portal (https://gdc.cancer.gov/) (*N* = 170), and TCGA transcript per million data (TPM) were computed from the recount2 dataset [18] (*N* = 169), as specified by Arora et al. [4] to be consistent with available Chinese Glioma Genome Atlas (CGGA) TPM data. The mutational status of *NOTCH1* and *PIK3CA* and somatic copy number alteration (SCNA) status of *CDKN2A* and chromosome arm 15q were determined from TCGA somatic mutation calls and SCNA GISTIC calls as previously described [56, 57]. Chromosome 15q loss was called if more than half of the genes on 15q were lost (Supplemental Fig. 1). C*DKN2A* homozygous deletions were called if *CDKN2A* had a GISTIC score of -2 in at least two of three versions of TCGA SCNA calls [56]. *MYC* activation was called as described in Kamoun et al. [38]. Characteristics including age, central nervous system (CNS) WHO grade, and overall survival were also downloaded from UCSC Xena, and chemotherapy and radiation therapy treatment details (postoperative_rx_tx, radiation_therapy) were downloaded from the GDC Data Portal. Treatment was considered adjuvant if it was labeled “ADJUVANT” in the metadata field “therapy_regimen” or was administered within 8 months of diagnosis (“pharmaceutical_tx_started_days_to”) and was the first treatment administered (Supplemental Fig. 2). Drug names were ascertained from the metadata field “pharmaceutical_therapy_drug_name.” Patients who received any adjuvant combination of procarbazine, lomustine (CCNU), and vincristine were considered treated by PCV, with the exception of EORTC 26951 Phase III trial data, where PCV refers to treatment with procarbazine, CCNU, and vincristine. TCGA IDH-mutational status and 1p/19q codeletion data were ascertained as previously described [56].

### TCGA histopathologic features

TCGA pathology reports for 156 oligodendroglioma patients were downloaded from the GDC Legacy Archive. Values for the Ki-67 proliferative index, presence of necrosis, presence of microvascular proliferation, and presence of mitotic figures were recorded. When the Ki-67 proliferative index was given as a range, we reported the upper bound. Ki-67 proliferative index values were considered high (≥ 10%) or low (< 10%) based on published reports [88]. We considered ≥ 6 mitoses per 10 high power fields (HPF) as high and < 6 mitoses per 10 HPF as low in line with WHO guidelines for tumor grading [44].

### TCGA radiographic features

The Multimodal Brain Tumor Segmentation Challenge (BraTS) 2020 training and validation datasets provide pre-operative, multimodal MRI tumor scans for 27 oligodendroglioma patients in the TCGA [6, 50]. Ground-truth segmentations delineating the boundaries of tumor edema, enhancement, and necrotic/non-enhancing tissue were available for 13 oligodendrogliomas. We used a segmentation machine learning model trained on the BraTS training set to assign segmentations to the remaining 14 oligodendrogliomas followed by manual correction [54, 55]. These segmentation maps were used to calculate the volume of each tumor compartment, which was then binarized as high or low (Supplemental Fig. 3).

As described in Bakas et al. [6], all multimodal MRI volumes contained pre- and post-contrast T1-weighted sequences, T2-weighted sequences, and T2 Fluid-Attenuated Inversion Recovery (FLAIR) sequences that were co-registered to the same anatomic atlas using affine registration provided by the Brain Linear Image Registration Tool (FLIRT) from the Functional Magnetic Resonance Imaging of the Brain Software Library (FSL) developed by the Oxford Center for Functional MRI [6, 34–36, 70, 85]. All MRI volumes were subsequently resampled to 1 mm^3^ isotropic voxel resolution and skull stripped by the Brain Extraction Tool (BET) from FSL or by Multi-Atlas Skull-Stripping [21] as described in Bakas et al. [6]. Resampling MRI volumes to the same resolution made direct comparisons of tumor volumes valid.

### Chinese glioma genome atlas (CGGA) dataset

Raw RNA gene counts for 48 CGGA [91] primary oligodendroglioma tumors were downloaded from recount2 [18] and used to compute TPM values [4]. Unsupervised dimension reduction [79] of CGGA and TCGA TPM data identified four CGGA oligodendroglioma outliers; these patients were excluded from our gene expression analysis (Supplemental Fig. 4). Clinical variables including age (*N* = 48), tumor CNS WHO grade (*N* = 48), and overall survival (*N* = 46) were deduced from published annotations [91].

### Capper et al. dataset

DNA methylation IDAT files from the Illumina 450 K platform for 170 oligodendroglioma tumors (119 primary) made available by the authors of Capper et al. [13] were downloaded from the NCBI Gene Expression Omnibus (GEO) under accession number GSE109381. Beta values were determined using the R package *minfi* [5]. Patient age for tumors (*N* = 160 total; *N* = 111 primary) and WHO tumor grade (*N* = 80 total, *N* = 61 primary) were determined from published clinical variables. Outcome data were not available. Tumors were considered oligodendroglioma if the classification of the version 11 Capper et al. random forest classifier was oligodendroglioma (O_IDH or O IDH) or if their WHO 2016 classification was Anaplastic oligodendroglioma, IDH-mutant and 1p/19q-codeleted or Oligodendroglioma, IDH-mutant and 1p/19q-codeleted. For age and WHO grade analyses, we limited our analyses on this dataset to primary tumors classified as oligodendroglioma by methylation classification and by WHO 2021 diagnostic criteria (*N* = 113).

### The glioma longitudinal analysis (GLASS) consortium dataset

The Synapse API was used to download gene expression TPM data (Primary *N* = 9; Total 19), DNA methylation beta values from the merged Illumina 450 K and EPIC platforms (beta.merged) (Primary *N* = 10; Total *N* = 24), as well as clinical variables age (*N* = 34), WHO grade (*N* = 34), and overall survival (*N* = 34) for oligodendroglioma tumors in the Glioma Longitudinal AnalySiS (GLASS) cohort (Data Release version 2021-11-16) [7, 19, 80]. GLASS tumors were determined to be oligodendrogliomas if the following conditions were met in the metadata for the patient’s primary tumor: "idh_status" was “IDHmut” and "codel_status" was "codel" or "idh_codel_subtype" was "IDHmut-codel." For our age and WHO grade analyses, we restricted our analyses to primary tumors whose "idh_codel_subtype" was "IDHmut-codel." All GLASS samples that appear in the TCGA were excluded.

### Jonsson et al. dataset

Published clinical variables, including overall survival and age for 84 primary oligodendroglioma tumors were downloaded from cBioPortal [15, 25, 37].

### Oligo nation

We downloaded metadata for 34 primary oligodendrogliomas with patient age annotations from the Oligo Nation cohort from PedcBioPortal (Data accessed 2024–02–06).

### EORTC 26951 phase III clinical trial

DNA methylation data and metadata from 115 WHO grade 3 diffuse gliomas made available by the authors of van den Bent, et al. [78] were downloaded from the NCBI GEO under accession number GSE48462. Patients were filtered to include only those who (1) harbored 1p/19q codeletions (according to 2021 WHO CNS tumor classification guidelines), (2) were treated by radiation therapy and procarbazine, CCNU, and vincristine, and (3) were profiled by Illumina 450 k arrays, which contain all *HOXD12* gene body probes we analyzed. After subsetting this cohort, only 10 tumors remained.

### Bulk gene RNA expression analysis

Differential gene expression analysis for bulk RNA-seq data was conducted using the R package *DESeq2* [45]. Genes shown in a volcano plot were limited to the genes that have raw gene counts data and TPM data available. When testing for gene expression prognostic implications during unbiased searches, we compared the outcomes of patients whose gene expression was above the median (elevated) to patients whose gene expression was equal or less than the median (low) based on the TPM values for the gene in question. Median HOXD12 expression was zero in the TCGA; thus, we considered HOXD12 expression elevated (HOXD12-postive) when it was non-zero and low otherwise (HOXD12-negative). Gene ontology analysis and gene set enrichment analysis were performed using the R package *clusterProfiler* [62] (R version 4.1.1).

### HOXD12-specific methylation analysis

We used public annotations to determine DNA methylation probe genomic regions (5′UTR, TSS -1500, TSS -200, gene body, or 3′UTR) [9]. The mean value of the beta values of the three probes (cg23130254, cg03964958, cg03371669) in *HOXD12’s* gene body was used to determine *HOXD12* hypermethylation. We determined a threshold by taking the mean value of this signature for patients with HOXD12-negative expression status (0.3034) and patients with HOXD12-positive expression status (0.4121) in the TCGA. Therefore, if a patient’s three gene body probe average exceeded this threshold (0.3577), they were considered *HOXD12* hypermethylated; otherwise, we considered that patient *HOXD12* hypomethylated.

### Blanco–Carmona et al. single cell dataset

We downloaded paired single-nucleus RNA sequencing (snRNA-seq) and single-nucleus ATAC sequencing (snATAC-seq) data for 7817 nuclei from the NCBI Gene Expression Omnibus (GEO) under accession number GSE205771 [10]. These data were available pre-processed in the Seurat R object “GSM6261343_oligodendroglioma_atac_seurat_object.rds.gz” [32, 72]. We used the precomputed UMAP embedding, and Seurat clusters published for these data, but we repeated the quality control described in Blanco-Carmona et al., which excluded 88 cells (*N* = 7729). We also recomputed cell types and tumor cell states and found results that differed from the published annotations. Specifically, the somatic copy number alterations (SCNAs) called by the R package inferCNV version 1.18.1 suggested that many of the nuclei reported as neoplastic by Blanco-Carmona et al. lacked 1p/19q codeletions [75]. Therefore, we used our inferCNV results for chromosome arms 1p and 19q to determine neoplastic nuclei, as described below.

### Wang et al. single cell dataset

Single-nucleus ATAC sequencing data (matrix, barcodes, peaks) for 10,732 nuclei were downloaded for two oligodendroglioma tumors—SF11612 (*N* = 5519) and SF11949 (*N* = 5213)—from the NCBI Gene Expression Omnibus (GEO) under accession number GSE138794 [82]. Quality control was performed separately on each tumor, yielding 10,281 nuclei (SF11612 *N* = 5323; SF11949 *N* = 4958). Single-nucleus RNA sequencing data was estimated using Signac’s function GeneActivity [73]. We then used the function merge to merge these datasets. Next, we normalized the merged, estimated snRNA-seq data using Seurat’s SCTransform. After this, we conducted dimension reduction using RunHarmony with 30 components. Following this, we used the functions RunUMAP, FindNeighbors, and FindClusters. These clusters were assigned non-neoplastic cell type and neoplastic cell state labels using SCNA calls from inferCNV and published gene markers as described below.

### Computational analysis of single cell datasets

As a first pass, gene markers were used to assign cell types. *RBFOX3* expression was used to identify neurons, *MBP*, *MOG*, and *CNP* expression were used to identify oligodendrocytes, *SLC1A2*, *GFAP*, *AQP4*, and *ALDH1L1* expression were used to identify astrocytes, and *PTPRC*, *CD163*, and *CD14* expression were used to identity microglia [76]. *SOX6* and *MAP2* expression were expected to be elevated in neoplastic nuclei, and *FLT1* expression was expected to be elevated in non-neoplastic nuclei compared to neoplastic nuclei. To more definitively identify tumor cells, we iteratively computed inferred SCNAs using the R package inferCNV version 1.18.1 with meta-cells, first using suspected non-neoplastic clusters as controls, which we updated after identifying 1p/19q codeletions in the output of inferCNV. Nuclei in clusters that showed universal loss of chromosome 1p and 19q and intact 1q and 19p were considered neoplastic. Clusters showing no evidence of 1p/19q codeletions were labeled with the cell type previously assigned by cell markers. Nuclei in clusters where the presence or absence of 1p/19q codeletions was not universal were excluded. We recomputed neoplastic cells for Blanco-Carmona et al. because we found instances where the published neoplastic nuclei labels and our inferCNV results contradicted each other.

We next assigned neoplastic nuclei to one of four metaprograms (oligodendrocyte progenitor cell (OPC)-like, Astro-like, Cycling, and ribosomal-enriched (RE)) as described in Blanco-Carmona et al. using the permutation test the authors detailed in their study [10]. We refer to these metaprograms as cell states. In our permutation test, we performed 100,000 permutations, which yielded a minimum possible *p*-value of 1−e5. For each nucleus, we computed a score for each cell state and an associated adjusted p-value (FDR) from our permutation test. We assigned each nucleus the cell state with the lowest FDR if the lowest FDR was less than a threshold of 0.0125; otherwise, we assigned the nucleus the cell state “gradient.” The threshold 0.0125 represented 0.05 divided by the number of cell states we considered. We calculated stemness scores and lineage scores for AC and OC exactly as described in Tirosh et al. [76].

### Pan-HOX methylation analysis

We pooled all TCGA (*N* = 171), Capper et al. (*N* = 170), and GLASS (*N* = 24) DNA methylation samples and subsetted the pooled dataset to gene body probes associated with genes in the HOXA, HOXB, HOXC, and HOXD loci. DNA methylation probe gene locations were deduced from Illumina 450 k metadata downloaded from USCS Xena and Illumina’s website. We found a total of 422 HOX gene body probes common between the TCGA, Capper et al., and GLASS cohorts. We batch-corrected samples in this pooled dataset using ComBat-seq to control for dataset source [90]. We subsequently trained 1000 UMAP models with different initializations and observed a clear, consistent separation of samples into two separated clusters. We excluded samples (*N* = 20) that switched clusters more than 10% of the time. Sample cluster membership was used to perform differential expression and differential DNA methylation. To compare the differential DNA methylation results of each HOX loci and individual HOX genes, we averaged the results of the probes associated with each HOX loci and each HOX gene.

### Statistical analysis

The Shapiro–Wilk test was used to test distribution normality. Hartigans' dip test was used to test distributions for unimodality. The log-rank test was used to test for univariate survival-association. We used Fisher’s exact test (Fisher’s) to assess statistical differences in binary data across two groups. Unless otherwise noted, we used the two-tailed Student's *t*-test when comparing groups of continuous values, except for patient age and Ki-67 proliferation index, for which we used the Mann-Whitney U Test due to concerns over distribution normality. The Pearson correlation coefficient was used to assess correlation. Cox’s proportional hazard (CPH) regression was used to compute hazard ratios (HR) and for multivariate survival analysis. Covariates whose variance inflation factor (VIF) indicated collinearity (VIF > 10) were omitted from CPH regression. The R package *rms* was used to construct a nomogram. Statistical analyses were performed in Python version 3.8.6 or R version 4.0.5 unless otherwise specified.

## Results

To study age and survival-associated molecular features of oligodendroglioma, we consulted 550 oligodendroglioma tumors from seven publicly available cohorts, including the TCGA, Chinese Glioma Genome Atlas (CGGA), Capper et al., Glioma Longitudinal AnalySiS Consortium (GLASS), Jonsson et al., Oligo Nation, and the EORTC 26951 Phase III clinical trial [7, 12, 13, 37, 41, 68, 77]. Patient age (*N* = 481), overall survival (*N* = 342), and bulk RNA-sequencing data (*N* = 232) from the TCGA, CGGA, and GLASS were used to nominate a molecular marker associated with an older, more aggressive subset of oligodendroglioma. The DNA methylation profile of this marker was subsequently analyzed in a group of 364 tumors from the TCGA, Capper et al., and GLASS cohorts and, along with RNA-sequencing data, subjected to association tests with features in 156 TCGA pathology reports and 27 pre-operative multimodal magnetic resonance imaging volumes. To isolate our analyses to neoplastic cells and interrogate proliferating phenotypes, we used single-nucleus RNA sequencing from 7817 nuclei and single-nucleus ATAC-sequencing data from 18,549 nuclei published by Blanco-Carmona et al. and Wang et al. [10, 82]. All data analyzed in this study are summarized in Supplemental Table 1.

### HOXD12 expression is age-associated and prognostic in oligodendroglioma

**Fig. 1:**
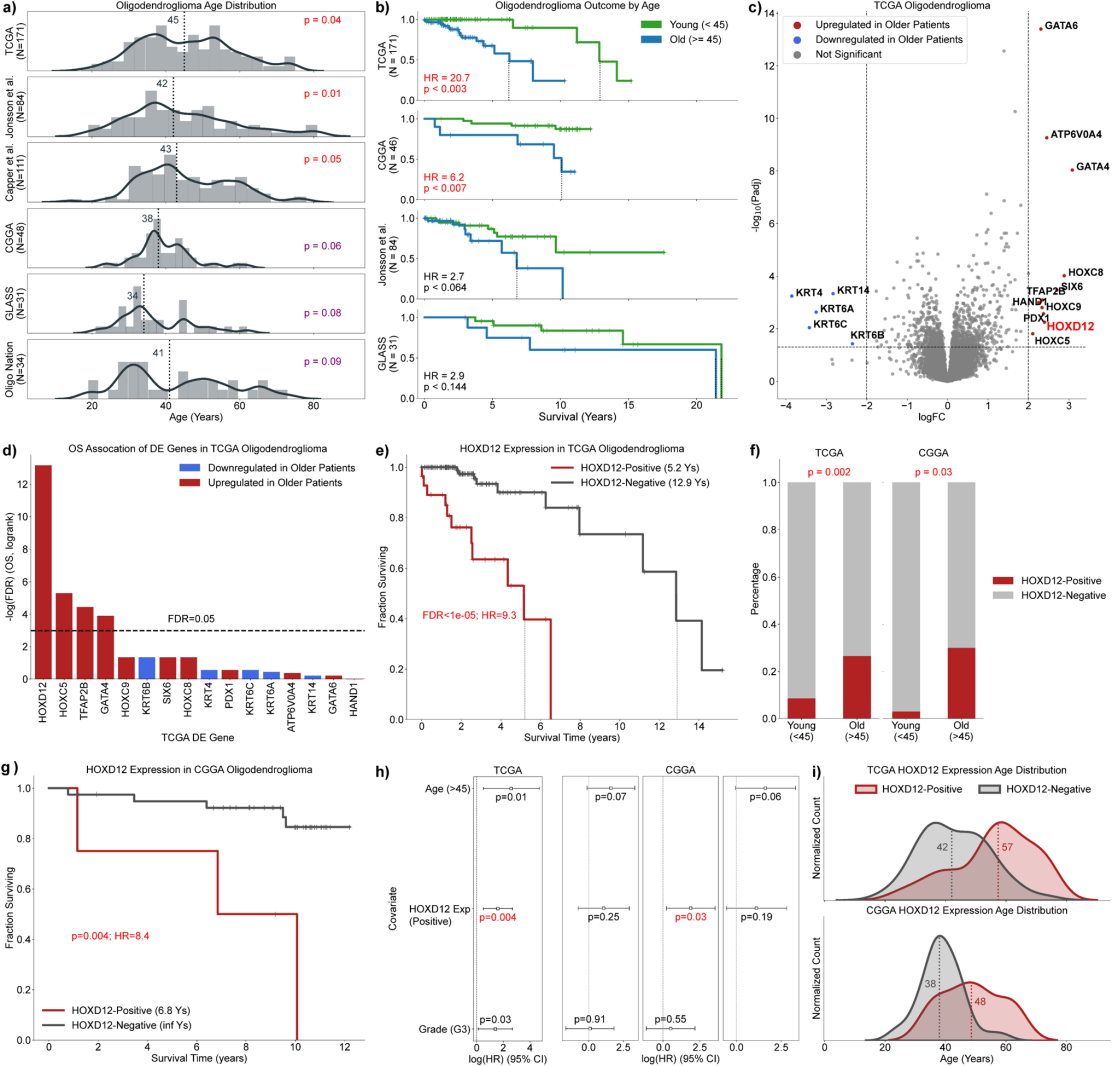
Investigation of the non-Gaussian age distribution in oligodendroglioma identifies HOXD12 expression as age and survival associated. **a** The distribution of oligodendroglioma patient age was significantly and marginally significantly non-Gaussian in six independent oligodendroglioma cohorts. **b** Older age was associated with worse outcomes in cohorts with survival data. **c** Unbiased differential gene expression analysis between older and younger patients returned 16 significantly differentially expressed genes, including several genes in the HOX, GATA, and keratin gene families. **d**, **e** Of the four differentially expressed genes that also stratified TCGA oligodendroglioma survival, HOXD12 had the strongest association with poor outcomes. **f** In addition to the TCGA, older oligodendroglioma patients were also enriched for HOXD12-positive expression status in the CGGA. **g** HOXD12-positive expression status was prognostic in the CGGA, validating the results from the TCGA. **h** HOXD12-positive expression status was prognostic independent of patient age and CNS WHO grade in the TCGA and independent of CNS WHO grade in the CGGA. **i** Age distributions of TCGA and CGGA oligodendrogliomas when partitioned by HOXD12-positive and HOXD12-negative expression status

Contrary to most cancer types, the age distribution of oligodendroglioma patients has been reported to appear bimodal, possibly indicating that certain age ranges are associated with different tumor biology [17, 64]. Although we were unable to statistically judge whether oligodendroglioma patient age distributions were multimodal in our data (Hartigans' dip test), we did find many cohorts exhibited a non-Gaussian distribution. Unlike IDH-wildtype glioblastoma and IDH-mutant astrocytoma adult-type diffuse gliomas in the TCGA, patient age in TCGA oligodendrogliomas was not normally distributed (*p* < 0.04, Shapiro–Wilk) (Supplemental Fig. 5). In addition to the TCGA, oligodendroglioma age was significantly or marginally significantly non-Gaussian distributed among primary tumors in cohorts published by Jonsson et al. (*N* = 84, *p* = 0.01, Shapiro–Wilk), Capper et al. (*N* = 111, *p* = 0.048), the CGGA (*N* = 48, *p* = 0.06), the GLASS Consortium (*N* = 31, *p* = 0.08), and Oligo Nation (*N* = 34, *p* = 0.09) (Fig. 1a) [7, 13, 19, 37, 80, 91]. In addition, all non-TCGA datasets were enriched for younger, longer surviving patients compared to the TCGA, suggesting that older patients may be underrepresented and obscure a clearer bimodal distribution in these datasets (Fig. 1b). Given that age is prognostic and non-Gaussian distributed in oligodendroglioma, we hypothesized that a distinctive, aggressive subtype of oligodendroglioma that occurs at older ages may exist.

To test genes for age and survival association, we first performed an unbiased differential gene expression analysis comparing older (≥ 45 years) and younger TCGA oligodendrogliomas and identified 16 differentially expressed genes. These genes included developmental transcription factors in the HOX and GATA gene families, which were upregulated in older patients, and keratin genes, which were downregulated in older patients (Fig. 1c). Gene ontology analyses of the results from this differential gene expression analysis showed that the most significantly activated pathways in older TCGA oligodendrogliomas were linked to developmental transcription factors and included DNA-binding transcription activator activity (*p*_adj_ < 1e−6) and the development of the embryonic skeletal system (*p*_adj_ < 1e−7), appendages (*p*_adj_ < 1e−6), and limbs (*p*_adj_ < 1e−6) (Supplemental Fig. 6a,b). Suppressed pathways included cornification (*p*_adj_ < 1e−13) and keratinization (*p*_adj_ < 1e−10) and other pathways that involve structural proteins that control cell shape. Our results were supported by a gene set enrichment analysis, whose most significant results were the suppression of cornification (*p*_adj_ < 1e−13) and the activation of embryonic skeletal system development (*p*_adj_ < 1e−7) (Supplemental Fig. 6c).

We followed these age-related analyses with survival analyses focused on the 16 differentially expressed genes identified above. To compare high and low gene expression levels, we compared patients with gene expression above a gene’s median expression level (elevated) to patients whose gene expression is below or equal to that gene’s median expression level (low). Of these 16 genes, 4 genes conferred significantly worse outcomes when elevated, of which *HOXD12* was the most significant and severe (HR = 9.3, FDR < 1e−5, log-rank) (Fig. 1d,e). Given that the median HOXD12 expression in the TCGA was zero, we refer to elevated HOXD12 expression as HOXD12-positive. HOXD12-positive expression status’ statistical associations with age and survival were validated in the CGGA (Fig. 1f,g). Older (≥ 45 years old) CGGA oligodendroglioma patients were significantly enriched for HOXD12-positive patients (*p* = 0.03, Fisher’s), and HOXD12-positive status was prognostic in the CGGA (*p* < 0.001, log-rank), incurring a similar hazard ratio (HR) as observed in the TCGA (TCGA HR = 9.3, CGGA HR = 8.4). In multivariate survival analyses, *HOXD12* was the only one of the four genes identified as prognostic whose elevated expression was also prognostic independent of patient age and WHO grade in the TCGA (FDR = 0.02, CPH) (Fig. 1h, Supplemental Fig. 7). HOXD12-positive status was independently prognostic of WHO grade in the CGGA but lost its significance when patient age was accounted for, possibly because the sample size (*N* = 44) was relatively small for a multivariate analysis. For both TCGA and CGGA cohorts, when HOXD12-positive and HOXD12-negative patients were considered separately, their age distributions more closely resembled Gaussian distributions (Fig. 1i). Similar trends toward age and survival association were found in the GLASS dataset; however, low patient count limited these analyses (*N* = 9) (Supplemental Fig. 8).

### HOXD12 gene body hypermethylation is age-associated and predictive of poor survival independent of HOXD12 expression

**Fig. 2:**
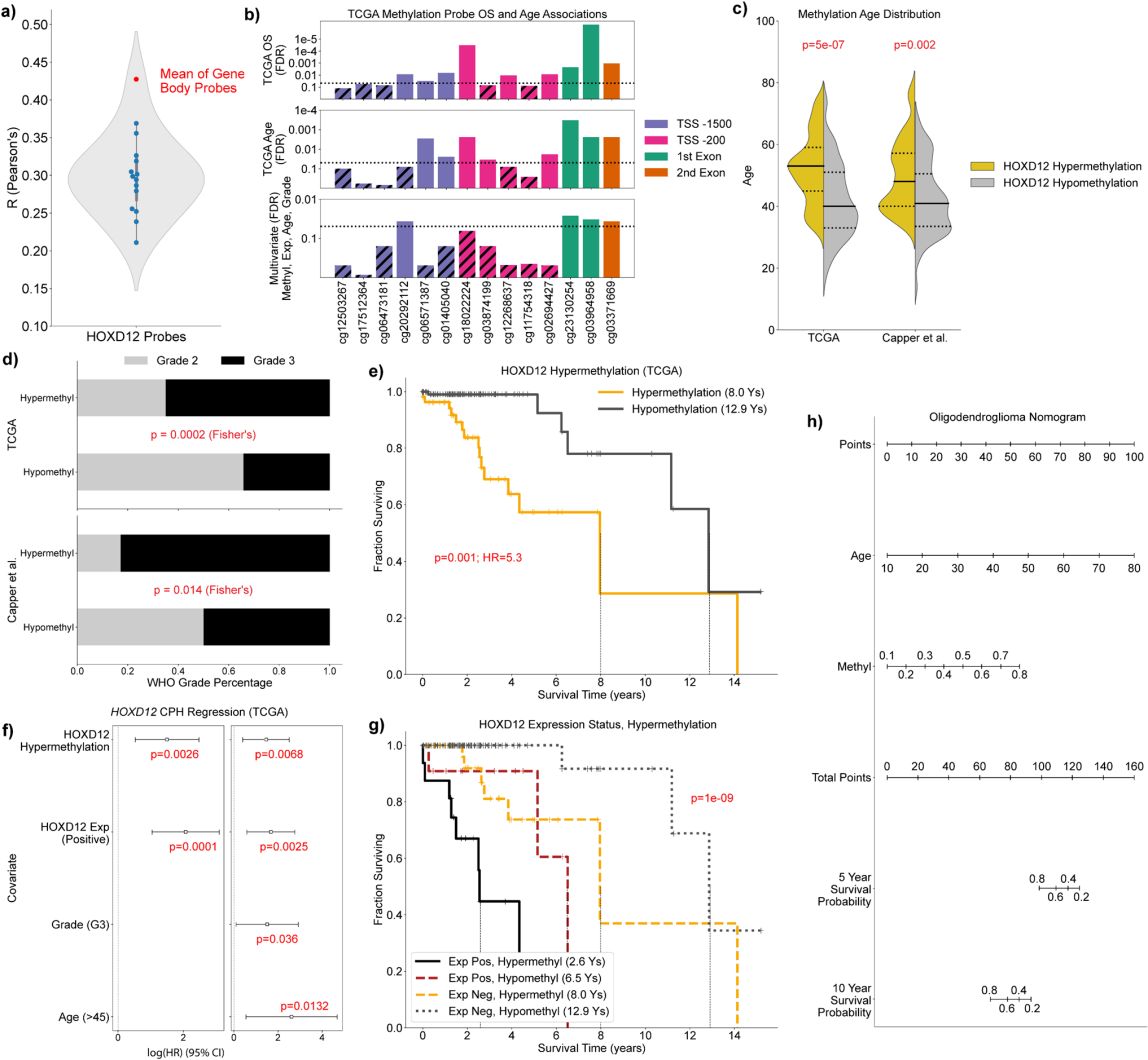
HOXD12 Hypermethylation is Associated with Age and Predictive of Poor Survival Independent of HOXD12 Expression. **a** All *HOXD12*-associated methylation probes (*N* = 14) were significantly positively correlated with *HOXD12* expression in the TCGA. **b** *HOXD12*-associated probes were tested for age, univariate survival, and multivariate survival associations in the TCGA using specified thresholds. Only the three probes located within *HOXD12’s* gene body passed all such tests. **c** *HOXD12* hypermethylation was associated with age in the TCGA and the Capper et al. cohort. **d** *HOXD12* hypermethylated oligodendrogliomas from Capper et al. and the TCGA were enriched for higher CNS WHO grade. **e** *HOXD12* hypermethylation was prognostic in the TCGA. **f** Nomogram for predicting 5- and 10-year overall survival using patient age, *HOXD12* methylation level, and CNS WHO grade. **g** Multivariate survival analyses showed *HOXD12* hypermethylation was prognostic independent of HOXD12-positive expression status, age, and CNS WHO grade. the TCGA. **h** Oligodendroglioma TCGA patients harboring HOXD12-positive expression status and *HOXD12* gene body hypermethylation had the worst outcomes (overall survival = 2.6 years)

Given that DNA methylation and gene transcription are related, we sought to evaluate the relationship between *HOXD12* methylation levels and patient age and survival. We found that every Illumina 450 k array DNA methylation probe associated with *HOXD12* (*N* = 14) was positively correlated with HOXD12 expression in the TCGA, suggesting that *HOXD12* hypermethylation was associated with higher transcriptional activity (Fig. 2a). Accordingly, we hypothesized that *HOXD12* hypermethylation would be more prevalent in older oligodendroglioma patients and associated with poor survival. To test this hypothesis, we defined a *HOXD12* methylation signature and set a threshold above which we deemed patients *HOXD12* hypermethylated. This signature was developed by testing each probe for age and survival association in both univariate and multivariate analysis (Methods) (Fig. 2b, Supplemental Fig. 9). Interestingly, the only probes that reached significance (*p* < 0.05) in all tests were *HOXD12*’s three gene body probes, which is consistent with the notion that gene body methylation is often positively correlated with gene expression and a report that *HOXD12* hypermethylation is linked to higher HOXD12 expression levels [69, 87]. To formally establish a *HOXD12* gene body hypermethylation threshold, we considered the mean of the beta values for the three *HOXD12* gene body methylation probes for patients with HOXD12-positive expression status and for patients with HOXD12-negative expression status. We chose the mean of these two values (0.3577) as the *HOXD12* gene body hypermethylation threshold.

Using this gene body methylation signature and threshold, we found that *HOXD12* gene body hypermethylation was associated with age in the TCGA (p < 1e-6, Mann–Whitney U) and Capper et al. cohorts (*p* = 0.002, Mann–Whitney U) (Fig. 2c, Supplemental Fig. 10). While this age association was not as strong in the Capper et al. cohort, this is likely because the Capper et al. cohort was disproportionally enriched for younger, WHO grade 3 tumors compared to all other datasets we analyzed (Supplemental Fig. 11). Furthermore, *HOXD12* gene body hypermethylation was prognostic in the TCGA in a univariate analysis (HR = 4.2, *p* < 0.001, log-rank) and two multivariate analyses, the first of which accounted for HOXD12 expression status (*p* < 0.003, CPH) and the second accounted for HOXD12 expression status, patient age, and WHO grade (*p* < 0.007, CPH) (Fig. 2d,e). Patients harboring HOXD12-positive expression status and *HOXD12* gene body hypermethylation in the TCGA formed the class of oligodendrogliomas with the worst outcomes, with a median overall survival of only 2.6 years (Fig. 2f). Although outcome data was not available for the Capper et al. cohort, oligodendrogliomas from this cohort were substantially enriched for central nervous system (CNS) WHO grade 3 tumors (*p* = 0.01, Fisher’s), as were their TCGA counterparts (*p* < 0.001, Fisher’s), indicating that *HOXD12* gene body hypermethylation was likely prognostic in the Capper et al. cohort (Fig. 2g). *HOXD12* gene body hypermethylation in the GLASS dataset showed similar patterns but suffered from a low sample count (*N* = 10) (Supplemental Fig. 12). To explore the clinical applicability of *HOXD12* gene body methylation, we developed a nomogram predicting 5- and 10-year overall survival using patient age, *HOXD12* gene body methylation level, and CNS WHO grade using TCGA data (Fig. 2h). Unfortunately, immunohistochemistry for HOXD12 was not a sufficiently sensitive surrogate marker to detect a difference between *HOXD12* gene body methylation levels in a small set of oligodendrogliomas (*N* = 10) (Supplemental Fig. 13).

### HOXD12-positive expression status and gene body hypermethylation are prognostic independent of key histopathologic, genomic, and radiographic features

**Fig. 3:**
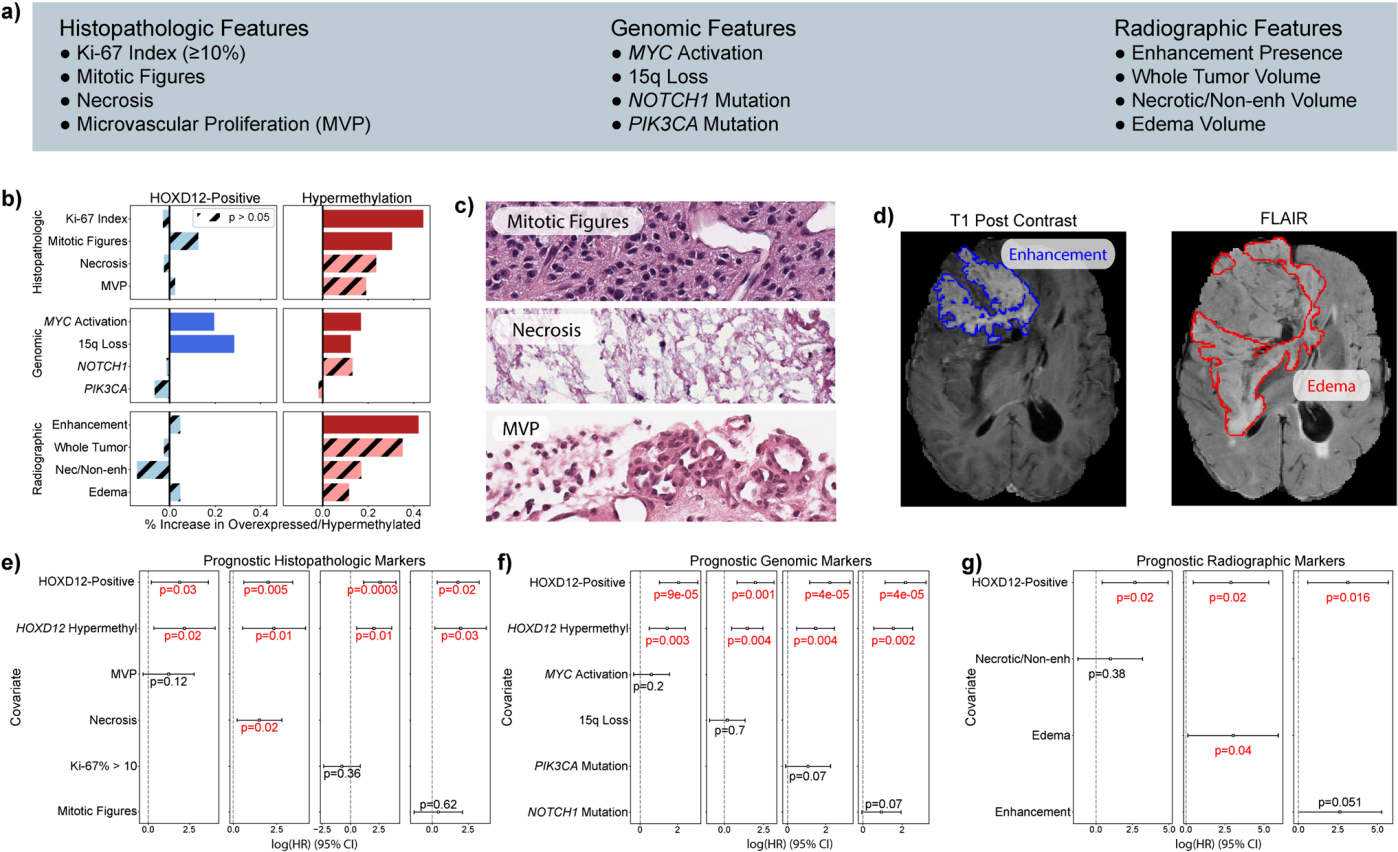
HOXD12-Positive Expression and Gene Body Hypermethylation are Independently Prognostic of Key Histopathologic, Genomic, and Radiographic Features. **a** List of key tested histopathologic, genomic, and radiographic features. **b** *HOXD12* gene body hypermethylation was linked to high Ki-67 proliferative index and the presence of mitotic figures. HOXD12-positive expression status and hypermethylation were associated with the loss of chromosome arm 15q and *MYC* activation. *HOXD12* hypermethylation was associated with the presence of T1-post-contrast enhancement. **c** Representative H&E-stained sections of a HOXD12-positive and gene body hypermethylated TCGA oligodendroglioma (TCGA-DU-7018) showed increased mitotic activity, necrosis, and MVP. **d** MRI of the same patient showed strong contrast enhancement (blue) and extensive peritumoral edema (red) on T1-post contrast and FLAIR MRI sequences, respectively. **e**, **f** HOXD12-positive expression status and hypermethylation were independently prognostic of all tested histopathologic and genomic variables. **g** HOXD12-positive expression status was independently prognostic of all tested radiographic features

Having shown that HOXD12-positive expression status and *HOXD12* gene body hypermethylation were markers for poor survival in oligodendroglioma, we interrogated their relationship with clinically relevant histopathologic, genomic, and radiographic features in the TCGA (Fig. 3a). Among histopathologic features, *HOXD12* gene body hypermethylation was linked to a high Ki-67 proliferative index (≥ 10%) (*p* < 0.0001, Fisher’s) and the presence of mitotic figures (*p* = 0.015, Fisher’s) but not to the presence of necrosis or microvascular proliferation (MVP) (Fig. 3b). Among proposed prognostic genomic alterations, we found that HOXD12-positive expression status and *HOXD12* gene body hypermethylation were associated with the loss of chromosome arm 15q (*p* < 0.001, *p* = 0.049, Fisher’s) and *MYC* activation (p = 0.04, p < 0.03, Fisher’s) but not with *NOTCH1* mutations or *PIK3CA* mutations. *CDKN2A* homozygous deletions were not tested because fewer than 1% (1/171) of TCGA oligodendrogliomas harbored these alterations. Radiographically, *HOXD12* gene body hypermethylation was associated with the presence of T1-post contrast enhancement in a set of 27 multimodal pre-operative MRIs from the TCGA (*p* = 0.03, Fisher’s), while HOXD12-positive expression status was not significantly associated with any tested radiographic feature. These associations suggest that tumors expressing HOXD12 and harboring *HOXD12* gene body hypermethylation are not only prognostic, but they also appear more aggressive histopathologically, molecularly, and radiographically. To highlight these features, we identified a representative tumor harboring both HOXD12-positive expression status and *HOXD12* gene body hypermethylation that displays mitotic figures, necrosis, and MVP (Fig. 3c), as well as contrast enhancement and substantial peritumoral edema (Fig. 3d).

Remarkably, HOXD12-positive expression status and *HOXD12* gene body hypermethylation were independently prognostic of all tested histopathologic and genomic variables (Fig. 3e, f). These multivariate analyses indicated that the statuses of HOXD12 expression and gene body methylation may be better indicators of outcome than histopathologic features used to determine CNS WHO grade (mitotic features, necrosis, MVP) and genomic markers nominated by other studies [2, 3, 30, 38, 74]. Similarly, HOXD12-positive expression status was independently prognostic of all testable radiographic features (total tumor volume omitted due to collinearity). However, *HOXD12* gene body hypermethylation was not (p = 0.12), possibly because the MRI dataset was small (Fig. 3g, Supplemental Fig. 14). Regrettably, it was not possible to validate our findings in the CGGA or Capper et al. cohorts because of insufficient imaging or histopathology data.

Importantly, the TCGA survival associations observed here were likely not due to treatment differences because the use of adjuvant chemotherapy (*N* = 152), predominately temozolomide, and/or adjuvant radiation therapy (RT) (*N* = 151) were not statistically associated with *HOXD12* gene body hypermethylation or HOXD12-positive expression status (Supplemental Fig. 15a, b, Supplemental Table 2). Of note, TCGA WHO grade 2 oligodendrogliomas were treated with postoperative chemoradiation dramatically less frequently than TCGA WHO grade 3 oligodendrogliomas (9% vs. 55%, *p* < 0.0001, Fisher’s) (Supplemental Fig. 15c). It is conceivable that if *HOXD12* gene body methylation status had been incorporated as a molecular risk factor, patients with *HOXD12* gene body hypermethylated tumors may have been treated more commonly with chemoradiation, as the case with WHO grade 3 tumors, and the survival differences we observed may have been slimmer.

To further control for confounding treatment differences and to test *HOXD12* gene body hypermethylation in a prospective cohort, we also analyzed DNA methylation data from the prospective EORTC 26951 Phase III trial] [41, 77]. EORTC 26951 Phase III compared RT to RT and six cycles of chemotherapy agents procarbazine, CCNU, and vincristine (PCV) in WHO grade 3 diffuse gliomas. For our analysis, we included EORTC patients who met the following three criteria: (1) their tumor met the current WHO CNS classification system definition of oligodendroglioma (including 1p/19q-codeletion), (2) they were treated with the trial’s uniform chemoradiation regimen (RT + PCV), and (3) their tumor had methylation data sufficient to determine *HOXD12* gene body methylation status. Unfortunately, most of the 115 tumors with profiled DNA methylation data were excluded because, unlike Illumina 450 k arrays, they were profiled with Illumina 27 k arrays, which lack two of the three *HOXD12* gene body probes in our *HOXD12* gene body methylation signature. Only 10 tumors met these three selection criteria, of which four were *HOXD12* gene body hypomethylated and six were *HOXD12* gene body hypermethylated. Our analysis of these data, although severely underpowered, did show a weak trend toward worse survival among *HOXD12* gene body hypermethylated patients in both overall survival (HR = 2.8, *p* = 0.36) and progression-free survival (HR = 2.8, *p* = 0.36) (Supplemental Fig. 16). Overall, more extensive prospective follow-up data and more feasible clinical testing are necessary to definitively establish any prognostic utility of HOXD12-positive expression status or *HOXD12* gene body methylation status.

### Increased HOXD12 activity in neoplastic cells is associated with a proliferative phenotype

**Fig. 4:**
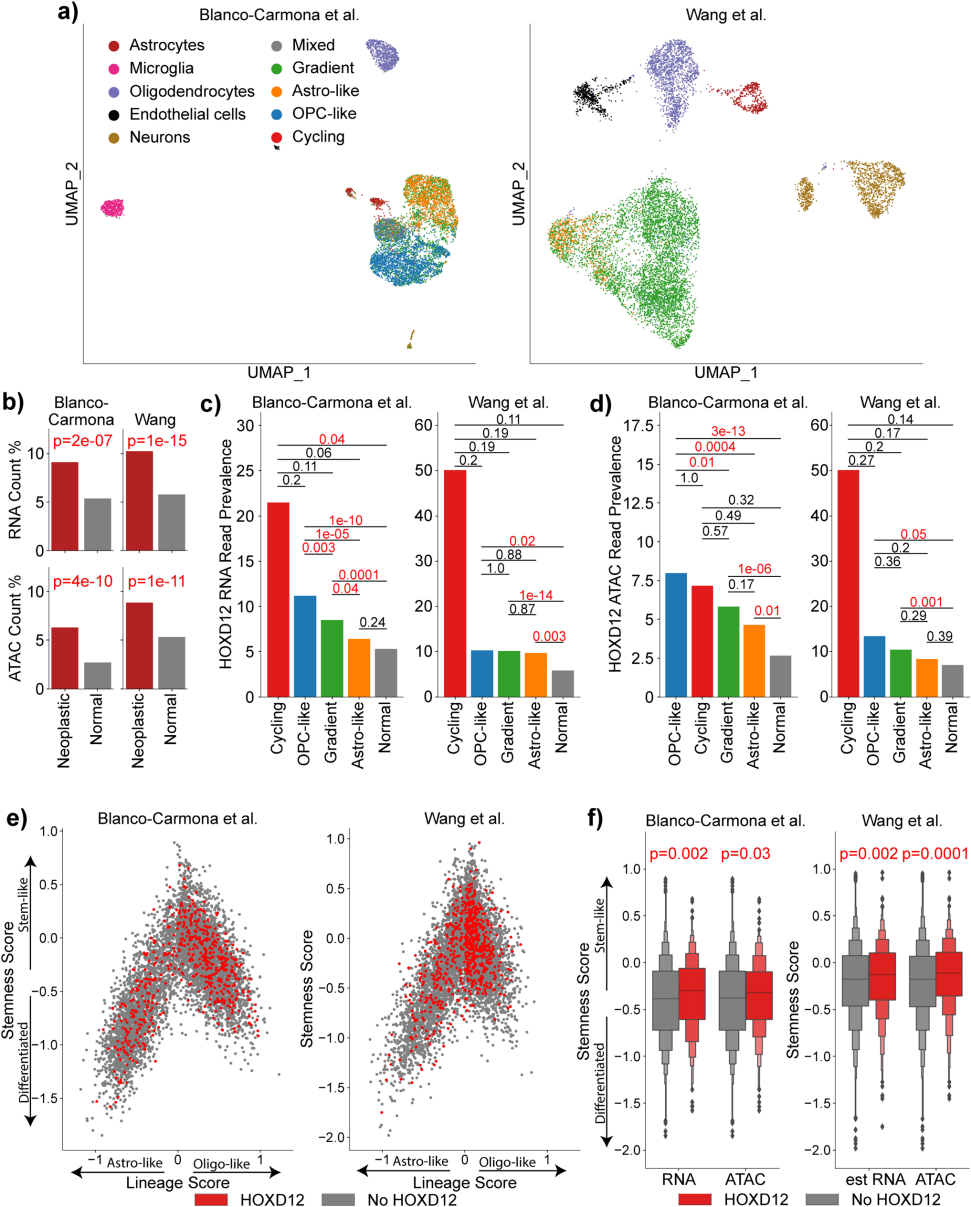
HOXD12 activity is associated with neoplastic nuclei, proliferating cells, and an increased stem-like phenotype. **a** UMAP embeddings of single-nuclei RNA-seq and single-nuclei ATAC-derived single-nuclei RNA-seq from the Blanco-Carmona et al. and Wang et al. cohorts, respectively, labeled by neoplastic cell state or non-neoplastic cell type. Neoplastic nuclei are labeled as Gradient, Astro-like, OPC-like, or Cycling. **b** In both the Blanco-Carmona et al. and Wang et al. cohorts, single-nucleus HOXD12 RNA reads and single-nucleus ATAC HOXD12 reads were more prevalent in neoplastic tissue compared to non-neoplastic tissue. **c**, **d** Among neoplastic nuclei, HOXD12 RNA and ATAC reads were most common in OPC-like and cycling cells, which are associated with stem-like and proliferative phenotypes. **e** Lineage and stemness scores for oligodendroglioma tumors nuclei in the Blanco-Carmona et al. and Wang et al. cohorts. **f** Nucleus stemness scores are higher in tumor nuclei that have HOXD12 RNA or ATAC reads than those without in both the Blanco-Carmona et al. and Wang et al.

To determine HOXD12 activity in specific cell compartments and associated cell states, we analyzed high-quality paired oligodendroglioma single-nucleus ATAC sequencing (snATAC-seq) and single-nucleus RNA sequencing (snRNA-seq) data published by Blanco-Carmona et al. (*N* = 7729) and snATAC-seq data published by Wang et al. (*N* = 10,281), from which we estimated snRNA-seq reads using Signac’s GeneActivity function [10, 73, 82]. To identify neoplastic nuclei, we used inferCNV to compute the somatic copy number alteration status of chromosome arms 1p and 19q to determine whether they were codeleted, a unique feature of oligodendroglioma cells; we used published gene markers to assign cell types to non-neoplastic nuclei (Fig. 4a, Supplemental Figs. 17, 18) [48, 53, 75, 81, 84]. Neoplastic cell states were assigned using lineage scores for oligodendrocyte progenitor cell (OPC)-like, Astro-like, Cycling, and ribosomal-enriched (RE) cell states for each nucleus, which we computed using published gene sets [10, 81]. Using a previously described permutation test, we calculated and corrected *p*-values which we used for final cell state assignment [10]. All RE cells were excluded due to a low sample count. Stemness scores and lineage scores for OC-like and AC-like cell states were also computed as previously described [76].

We found that HOXD12 expression was significantly more common in cycling/proliferative neoplastic cells than in non-proliferative neoplastic cells, which, in turn, had a higher rate of HOXD12 expression than non-neoplastic brain cells. In general, HOXD12 expression and chromatin accessibility were significantly more prevalent in neoplastic nuclei compared to non-neoplastic nuclei in the Blanco-Carmona et al. and Wang et al. datasets (Fig. 4b). After cycling/proliferative neoplastic cells, HOXD12 snRNA-seq expression was most prevalent in OPC-like neoplastic cells in both datasets, followed by gradient and astro-like neoplastic cells (Fig. 4c). Likewise, snATAC-seq confirmed that neoplastic cells classified as cycling or OPC-like had the highest prevalence of HOXD12 chromatin accessibility followed by cells classified as gradient or astro-like (Fig. 4d). Given that cycling cells are in the process of dividing and that OPC-like cells are known for their proliferative potential and stem-like properties, we computed stemness scores for all neoplastic cells. For both snRNA-seq and snATAC-seq data, and in both the Blanco-Carmona et al. and Wang et al. datasets, nuclei with HOXD12 activity had significantly higher stemness scores (Student’s *t*-test) (Fig. 4e,f). Altogether, these single-nuclei data suggested that HOXD12 activity is associated with a proliferative phenotype in neoplastic cells and indicated that our previous analyses of bulk sequencing data reflect biological processes within the neoplastic cell compartment, rather than the non-neoplastic cells of the tumor microenvironment.

### A Pan-HOX methylation signature, driven by HOXD12, is associated with increased proliferation and poor outcomes

**Fig. 5:**
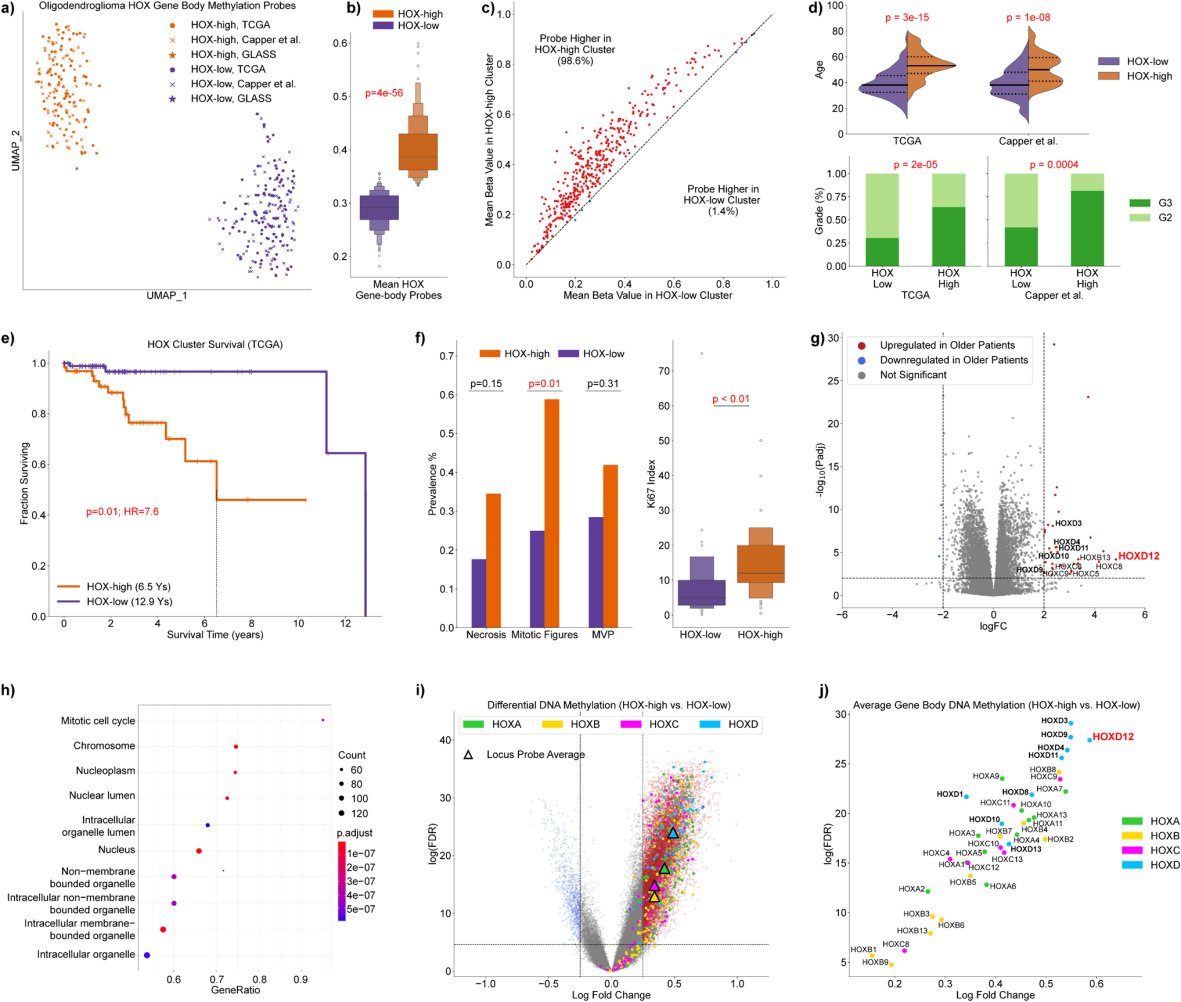
A Pan-HOX signature led by HOXD12 is associated with poor outcomes and proliferation. **a** A UMAP embedding of all HOX gene body associated DNA methylation probes for oligodendroglioma patients in the TCGA, Capper et al., and GLASS datasets (*N* = 365) formed two clear clusters termed HOX-high and HOX-low. **b** The mean value of HOX gene body probes is significantly higher in the HOX-high cluster than in the HOX-low cluster. **c** The mean value of 98.4% of HOX gene body probes is higher in the HOX-high cluster than in the HOX-low cluster. **d** The HOX-high cluster is associated with patient age and tumor WHO grade in the cohorts of the TCGA and Capper et al. Differences in TCGA and Capper et al. age distributions are likely attributable to the abundance of young, WHO grade 3 oligodendrogliomas in the Capper et al. cohort. **e** The TCGA patients in the HOX-high cluster harbor significantly worse outcomes than those in the HOX-low cluster. **f** TCGA tumors in the HOX-high cluster more commonly had mitotic figures and high Ki67 index; the presence of palisading necrosis and MVP were also elevated. **g** In a differential gene expression analysis between the two methylation classes, HOXD12 had the highest RNA expression log fold change. **h** TCGA patients in the HOX-high cluster were associated with the mitotic cell cycle pathway in a gene ontology analysis. **i** In a differential methylation analysis, gene body probes on the HOXD locus had higher log fold change on average compared to gene body probes from other HOX loci. **j** In this differential methylation analysis, gene body probes associated with HOXD12 had the highest average log fold change compared to other HOX genes

To place our HOXD12-centric findings in the context of all HOX genes, we analyzed the HOX gene body DNA methylation probes present in all 365 oligodendroglioma tumors from the TCGA (*N* = 171), Capper et al. (*N* = 170), and GLASS (*N* = 24) datasets. Two distinct, consistent clusters, not related to dataset membership, appeared across 1000 UMAP projections of these data generated using different initializations (Fig. 5a, Supplemental Fig. 19). We restricted our analysis to samples that remained in the same cluster in at least 90% of the UMAP projections (*N* = 345) (Supplemental Fig. 20). These two clusters were characterized by global HOX gene body DNA methylation levels: nearly all HOX-associated gene body probes had higher beta values in one cluster (HOX-high) compared to the other (HOX-low) (Fig. 5b, c). Patients in the HOX-high group were significantly older in the TCGA (*p* = 3e−15, Mann–Whitney U) and Capper et al. (*p* = 1e−8, Mann–Whitney U) datasets, and they disproportionally harbored higher CNS WHO grade (*p* = 2e−5, *p* = 0.0004, Fisher’s) (Fig. 5d, Supplemental Fig. 21). TCGA patients in the HOX-high group harbored more aggressive tumors, measured both by clinical outcomes and histological features. HOX-high TCGA patients suffered worse outcomes than those in the HOX-low group (HR = 7.6, *p* = 0.01). HOX-high TCGA tumors also more commonly had increased mitotic figures (*p* = 0.01, Fisher’s) and higher Ki-67 proliferative index (*p* < 0.01, Mann–Whitney U) (Fig. 5e, f). The presence of palisading necrosis and microvascular proliferation (MVP) were also elevated in HOX-high TCGA patients; however, their difference was less significant. Although the sample size of stable samples in the GLASS dataset was small (*N* = 9), we observed more movement from the HOX-low cluster to the HOX-high cluster as tumors evolved than vice versa (Supplemental Fig. 22a–c). The scarcity of these data made conclusions impossible to draw with confidence; however, GLASS samples in the HOX-high cluster tended to be recurrent, higher WHO grade, and shorter-lived (Supplemental Fig. 22d–f). As was the case with *HOXD12* gene body hypermethylation and HOXD12-positive expression status, HOX-high survival associations were not linked to lower rates of adjuvant therapy in the TCGA (Supplemental Fig. 23). In fact, the only significant associations we found were higher rates of adjuvant chemotherapy (p = 0.01, Fisher’s) and chemoradiation (*p* < 0.05) in HOX-high patients.

Next, we performed unbiased differential gene expression and differential DNA methylation analyses by comparing patients in the HOX-high group to patients in the HOX-low group. Remarkably, *HOXD12* was the gene with the highest RNA expression log-fold change between the HOX-high and HOX-low TCGA patients (4.8, *p*_adj_ < 7e−5) (Fig. 5g). Gene ontology analyses of the results from our differential gene expression analysis on TCGA oligodendroglioma tumors showed that the most significantly activated pathways in HOX-high patients were related to mitotic activity and cell cycle (*p*_adj_ < 1e−6) (Fig. 5h). In our differential DNA methylation analysis, gene body probes from the HOXD locus had more significant, higher log-fold change on average compared to gene body probes from other HOX loci (Fig. 5i). Among HOX genes, gene body probes associated with HOXD12 had the highest average log-fold change compared to gene body probes associated with other HOX genes (Fig. 5j, Supplemental Fig. 24). In general, gene body DNA methylation of HOX genes partitioned oligodendroglioma into two groups with lower and higher HOX gene body DNA methylation levels. The group of oligodendroglioma tumors with high HOX gene body DNA methylation levels was associated with poor survival, older patients, and more aggressive histology and was best differentiated from the HOX-low group by genes in the HOXD locus, especially *HOXD12*.

## Discussion

Although patients diagnosed with oligodendroglioma typically have better prognoses than their IDH-mutant astrocytoma and IDH-wildtype glioblastoma counterparts, oligodendroglioma patients have more variable outcomes, which underscores the need to make therapy intensities proportionate to individual patient risk. However, assessing a patient’s risk is difficult as only a handful of oligodendroglioma risk factors have been proposed to date, notably including patient age [2, 3, 30, 38, 74]. Unlike other adult-type diffuse gliomas, the age distribution of oligodendroglioma patients is non-Gaussian and reportedly bimodal, suggesting that investigations into age-associated biological differences in oligodendroglioma tumors are merited [17]. In this study, we sought to discover molecular markers associated with age and patient survival to gain insight into the relationship between patient age and oligodendroglioma tumor biology. We propose that HOXD12 expression and gene body hypermethylation are molecular features associated with poor prognoses that may be linked to two age-associated subtypes of oligodendroglioma.

HOXD12 expression was identified in an unbiased manner by filtering for genes whose expression was associated with age and survival in the TCGA. In addition to HOXD12 expression, we also found that *HOXD12* gene body hypermethylation was correlated with HOXD12 expression levels. Remarkably, *HOXD12* gene body hypermethylation was prognostic independent of HOXD12 expression, as well as patient age and tumor WHO grade. Our results were validated in oligodendroglioma cohorts published by the CGGA, Capper et al., and GLASS. We also showed *HOXD12* gene body hypermethylation and elevated expression were independently prognostic of published biomarkers and standard histopathological features, and we used single-nucleus RNA and ATAC sequencing to show that HOXD12 activity was localized to neoplastic cells rather than cells of the tumor microenvironment and was particularly strong within cycling and OPC-like cells. A pan-HOX DNA methylation analysis revealed an age and survival-associated HOX-high signature that was tightly associated with *HOXD12* gene body methylation. Overall, elevated HOXD12 expression and gene body hypermethylation were associated with an older, atypically aggressive subtype of oligodendroglioma. We propose that future biomarker-driven clinical trials of oligodendroglioma collect *HOXD12* gene body methylation data, possibly through whole-genome DNA methylation profiling. If *HOXD12* gene body methylation’s prognostic value is confirmed in trials such as these, *HOXD12* gene body methylation testing may be considered for risk-stratification in clinical, surgical neuropathology, as whole-genome DNA methylation array profiling has made its way into routine clinical practice across several academic medical centers [14, 24, 33, 39, 60, 61, 63, 65–67, 86].

*HOXD12* is a gene in the HOX gene family located on the HOXD locus on chromosome 2—one of four HOX loci (HOXA, HOXB, HOXC, and HOXD) found on chromosomes 7, 17, 12, and 2, respectively. In healthy individuals, HOX genes are involved in embryonic brain development and are usually absent in the adult brain; however, abnormal HOX gene expression and regulation have been extensively documented in cancer [1, 8, 29, 59]. Numerous genes in the HOX gene family have been reported to be involved in tumors of the CNS (as reviewed by Gonçalves et al. [29]), particularly in glioblastoma [16, 20, 22, 27, 29, 40, 42], primarily as oncogenes. More recently, HOX genes in the HOXA and HOXD loci have been implicated in IDH-mutant adult-type diffuse glioma progression and aggression [46, 47]. A study by Mamatjan et al. analyzed HOX gene overexpression and hypermethylation and proposed a seven HOX gene signature (HOXA4, HOXA7, HOXA10, HOXA13, HOXD3, HOXD9, and HOXD10) that was shown to be prognostic in IDH mutant astrocytoma and oligodendrogliomas [47]. The gene HOXD13 has also been implicated in glioma progression in a ChIP-seq analyses of 14 IDH-mutant gliomas, but this phenotype was not validated in oligodendroglioma [46].

Our findings support and extend the existing literature regarding oligodendroglioma heterogeneity. Kamoun et al. identified three subtypes of oligodendroglioma from four microarray datasets, of which the largest subset was negatively prognostic in the TCGA, weakly associated with age, and characterized by *MYC* activation [38]. Interestingly, we showed that TCGA patients that express HOXD12 or harbor *HOXD12* gene body hypermethylation were associated with age and *MYC* activation, but HOXD12 expression and gene body hypermethylation were prognostic independent of both. This suggests that HOXD12 expression and gene body hypermethylation may explain the reported prognostic value of *MYC* activation. A single-cell RNA-seq study by Tirosh et al. also interrogated oligodendroglioma molecular heterogeneity, albeit in an intra-tumoral setting, and linked neurodevelopmental transcription factor expression to a potentially proliferative stem/progenitor program in a small subpopulation of oligodendroglioma cells [76]. Our results corroborate these findings: HOXD12 is a neurodevelopmental transcription factor, HOXD12 expression was associated with a stemlike phenotype in single-nucleus sequencing data, and HOXD12 was expressed in a minority of neoplastic nuclei. The results of the study by Mamatjan et al. also align with our findings. Our unbiased pan-HOX analysis showed DNA methylation probes associated with HOX genes from the HOXA and HOXD loci were most differentially methylated between the prognostic HOX-high and HOX-low clusters. The unbiased nature of our analyses also boosts this study, as does our deep interaction of HOXD12 in multiple datasets and the supportive single-nucleus data we presented.

We do acknowledge several limitations in our study. Our analyses were conducted on retrospective data, which can suffer from sparse annotations, masked biases, and outdated treatment. To address these limitations, we confirmed our major results on at least two datasets, and we verified that there were no significant differences in treatment that would explain survival differences between compared groups in the TCGA. Another obstacle was our inability to detect HOXD12 by immunohistochemistry on a small set of patients; this must be further investigated before HOXD12 activity can be considered clinically relevant. Overall, more prospective human data must be collected and subjected to long-term follow-up to confirm any findings and subsequent use in risk stratification and clinical decision-making. Additionally, given the clinical benefit of IDH inhibitors in patients who have delayed chemoradiation, future study of *HOXD12* in the context of mutant IDH inhibition is merited and may assist in guiding clinical decisions [49, 52, 71].

## Supplementary Information

Below is the link to the electronic supplementary material.

Supplementary file1 (PDF 2842 kb)

Supplementary file2 (XLSX 12 kb)
