# Supplementary material for: HOXD12 defines an age-related aggressive subtype of oligodendroglioma
Source: Acta Neuropathol. 2024 Sep 11;148(1):41. doi: 10.1007/s00401-024-02802-1 (PMC11390787; doi:10.1007/s00401-024-02802-1)
Supplement: Supplementary file 1 — Supplementary file1 (PDF 2842 kb) [file 401_2024_2802_MOESM1_ESM.pdf]

SUPPLEMENTARY MATERIALS AND METHODS

NIH Oligodendroglioma Samples, Methylation, and Immunohistochemistry

An institutional search was performed to identify oligodendroglioma samples that have undergone whole-genome methylation profiling as a part of the routine clinical workup at the NIH Cancer Center and that additionally had sufficient remaining formalin-fixed paraffin-embedded (FFPE) tissue for immunohistochemistry. Ten such samples were identified. Whole-genome methylation array profiling was performed as previously described[1], and the resultant IDAT files were analyzed similarly to the other datasets in the main manuscript text. Immunostaining was performed on FFPE tissue using a polyclonal anti-HOXD12 antibody (Atlas Antibodies, Stockholm, Sweden) at 1:35 dilution on an automated stainer (Leica Bond-Max, Buffalo Grove, IL). Antibody binding was visualized using a ready-to-use detection kit (Leica Biosystems, Buffalo Grove, IL) with chromogen-labeled 3,3′-diaminobenzidine (DAB). HOXD12 stained-slides were reviewed by a board-certified neuropathologist (PJC) who was blinded to *HOXD12* methylation status. Human tissue use was approved by the NIH Institute Review Board.

SUPPLEMENTARY REFERENCES

1. Pratt D, Abdullaev Z, Papanicolau-Sengos A, et al. High-grade glioma with pleomorphic and pseudopapillary features (HPAP): a proposed type of circumscribed glioma in adults harboring frequent TP53 mutations and recurrent monosomy 13. *Acta Neuropathol.* 2022; 143(3):403-414.

SUPPLEMENTARY FIGURES

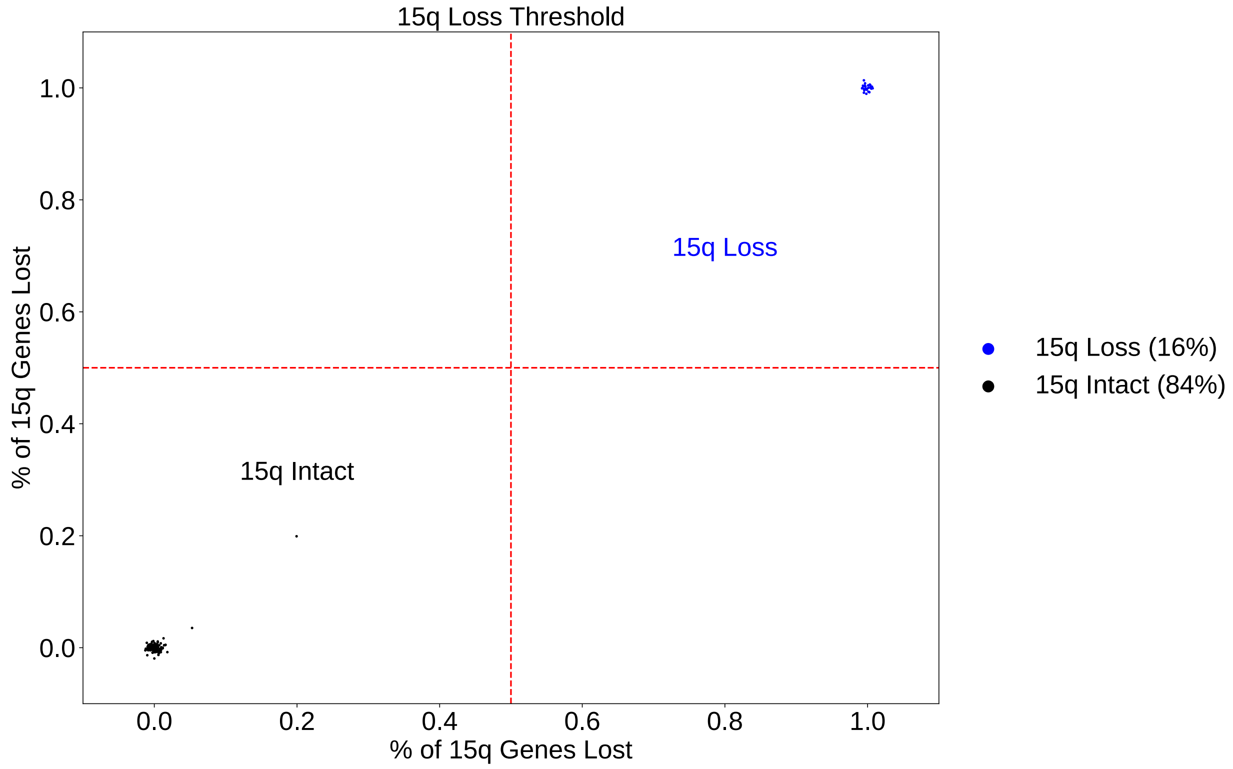

SUPPLEMENTAL FIGURE 1. 15q loss threshold. A 15q loss threshold of 50% gene loss clearly separates tumors with and without 15q loss. Noise is added to visualization to separate points.

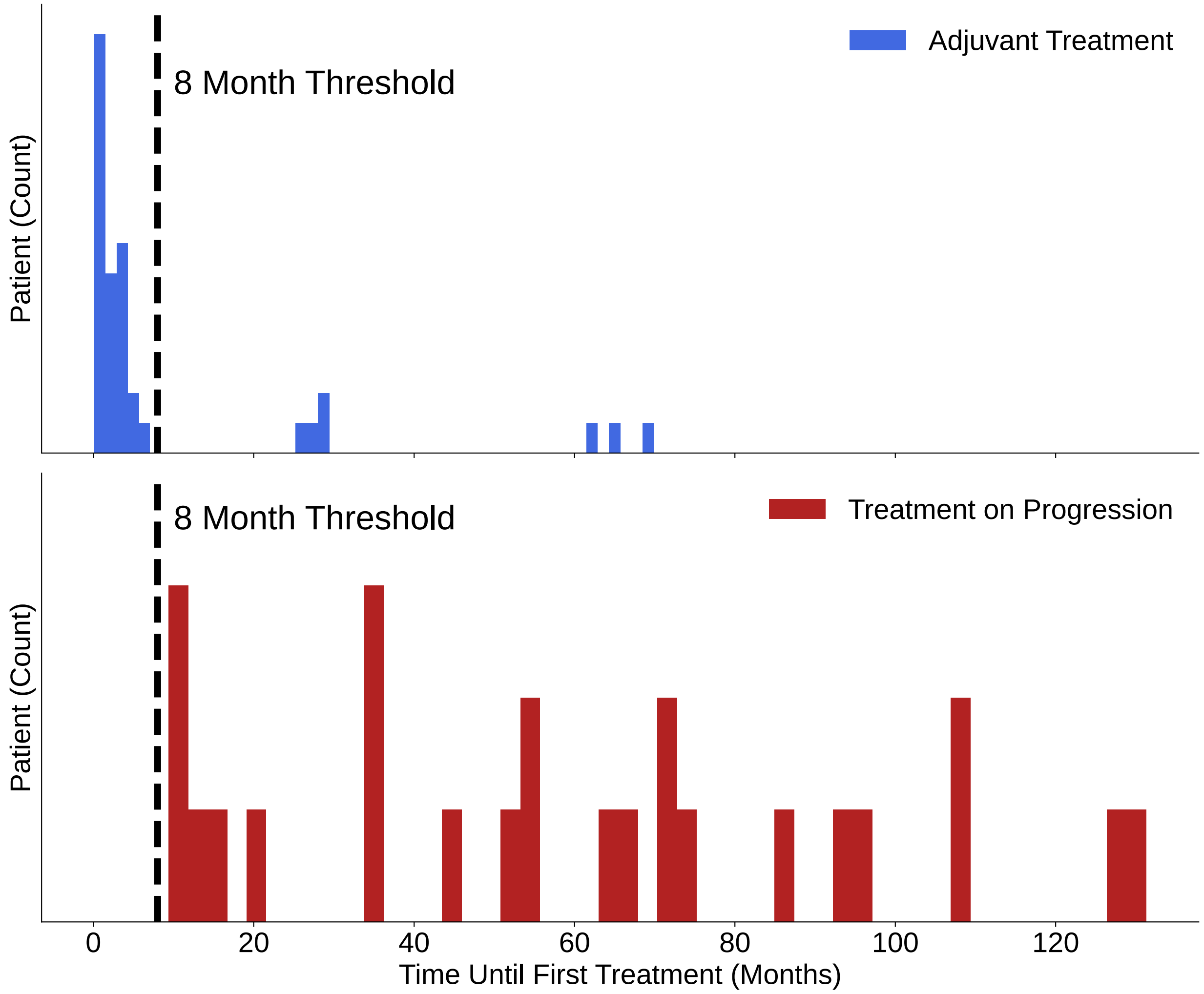

SUPPLEMENTAL FIGURE 2. Determination of adjuvant treatment. Treatment was considered adjuvant if it was labeled “ADJUVANT” in the metadata field “therapy_regimen” (blue) or was administered within eight months of diagnosis and was the first treatment administered. This threshold was chosen because no patients received treatment for progressive tumors earlier than eight months.

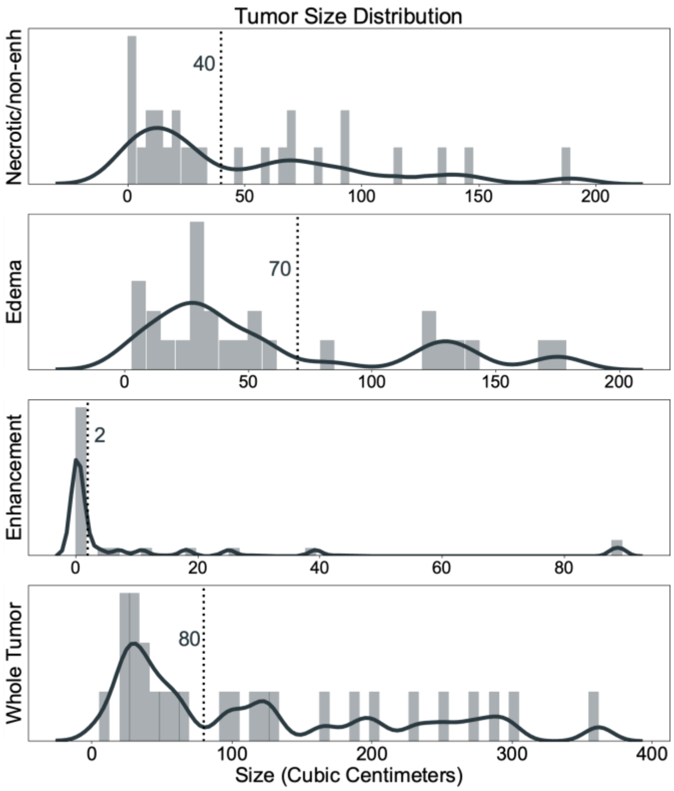

SUPPLEMENTAL FIGURE 3. MRI tumor compartment thresholds were determined visually. The volume of oligodendroglioma tumor compartments was binarized using visually determined thresholds. These thresholds were used to classify the necrotic/non-enhancing compartment, tumor edema compartment, and whole tumor volume as small or large. Tumor enhancement was classified as present (> 2 cm^3) or absent (< 2 cm^3).

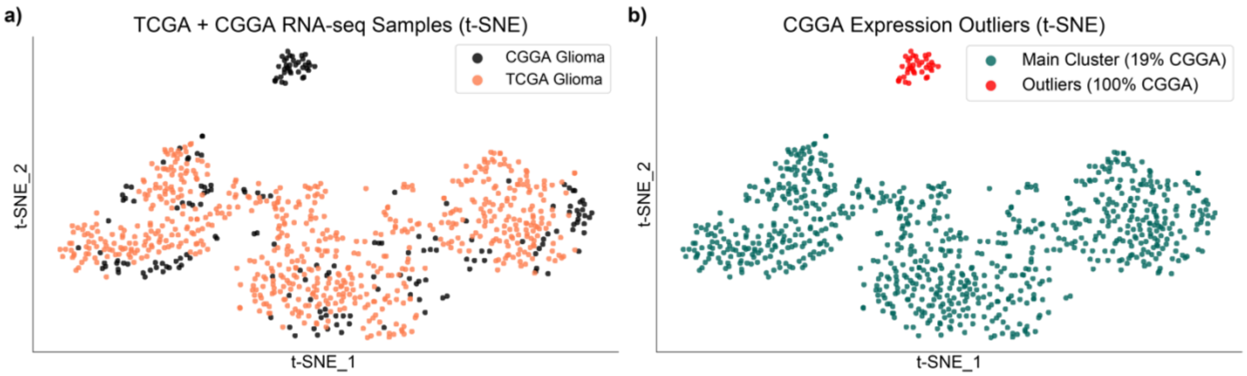

SUPPLEMENTAL FIGURE 4. CGGA RNA-seq data contains outliers. a) Unsupervised dimension reduction (t-SNE) of TCGA and CGGA RNA-seq TPM data computed from recount2 data shows that the TCGA and CGGA projection forms two distinct clusters. **b)** The smaller cluster consists of only CGGA patients, of which four are oligodendroglioma, IDH-mutant and 1p/19q-codeleted. We deemed these four tumors to be outliers because they appear dissimilar to all TCGA samples. It is unlikely that this cluster is the product of bioinformatic processing as all TCGA and CGGA TPM values were calculated using exactly the same pipeline.

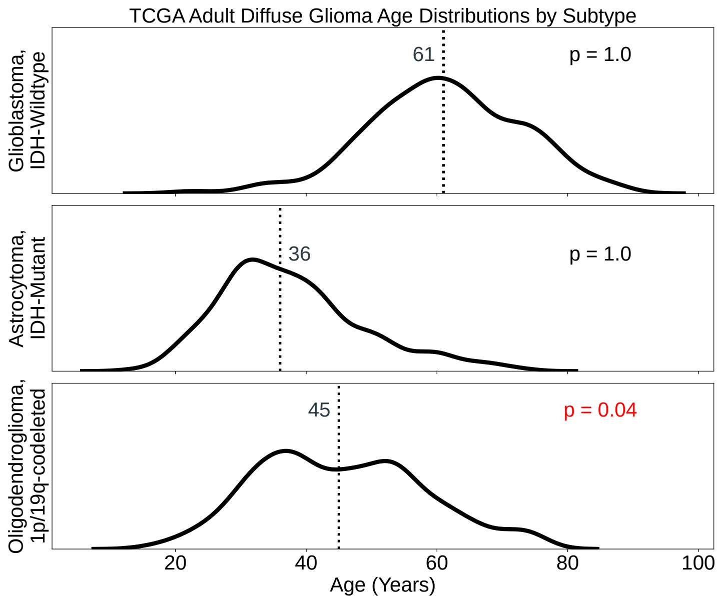

SUPPLEMENTAL FIGURE 5. Age distribution of TCGA adult-type diffuse gliomas. Unlike IDH-wildtype glioblastoma and IDH-mutant astrocytoma adult-type diffuse gliomas in the TCGA, patient age in TCGA oligodendrogliomas was not normally distributed (Shapiro-Wilk).

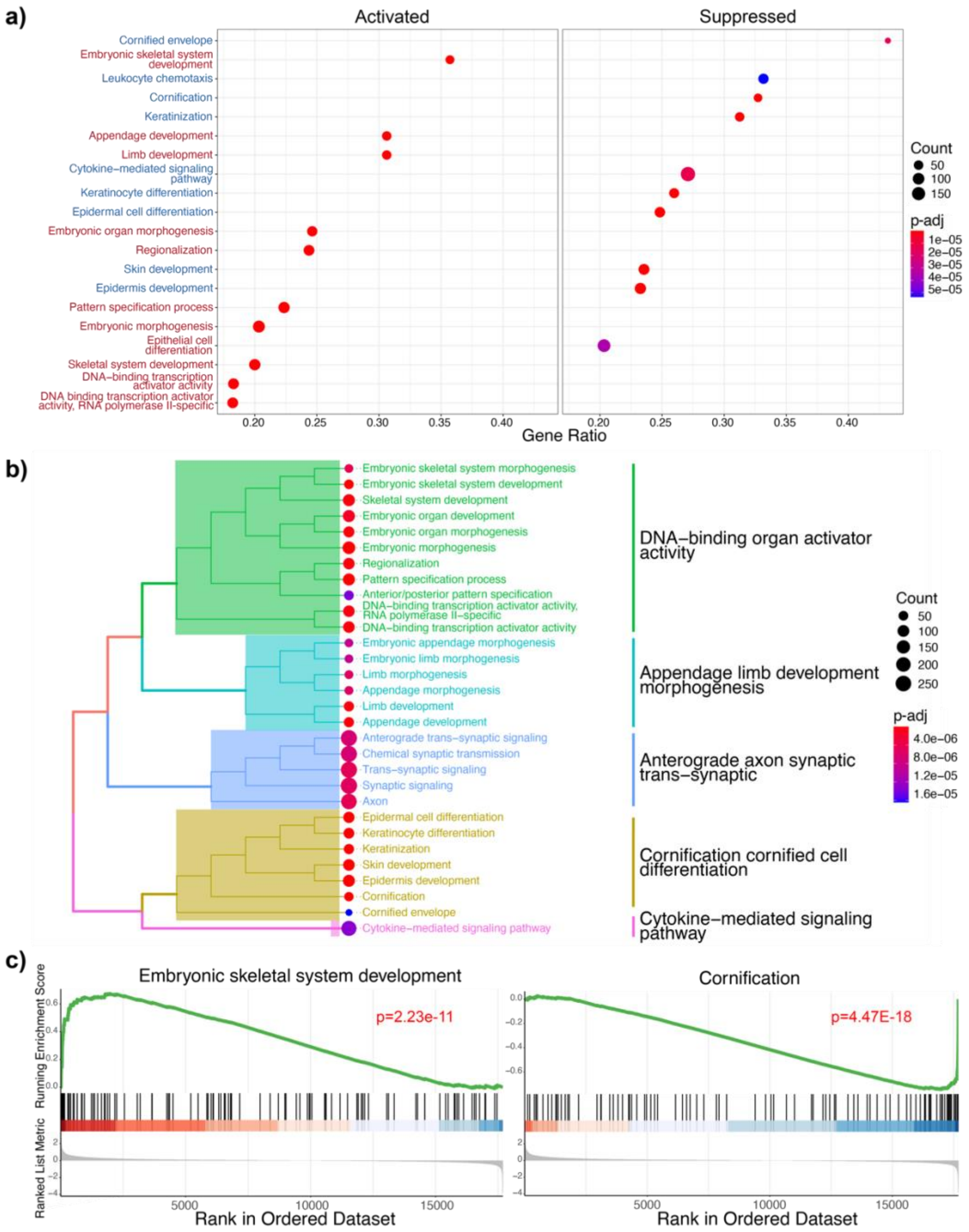

SUPPLEMENTAL FIGURE 6. Older Oligodendrogliomas are Associated with Increased Developmental Transcription Factors and Decreased Keratins. a, b) Gene ontology analysis showed that activated pathways in TCGA oligodendrogliomas from older patients were linked to developmental transcription factors, specifically DNA-binding transcription activator activity and the development of the embryonic skeletal system, appendages, and limbs. Suppressed pathways included cornification, keratinization, and other pathways that control cell shape. **c)** The top hits of gene set enrichment analyses were the activation of embryonic skeletal system development and suppression of cornification.

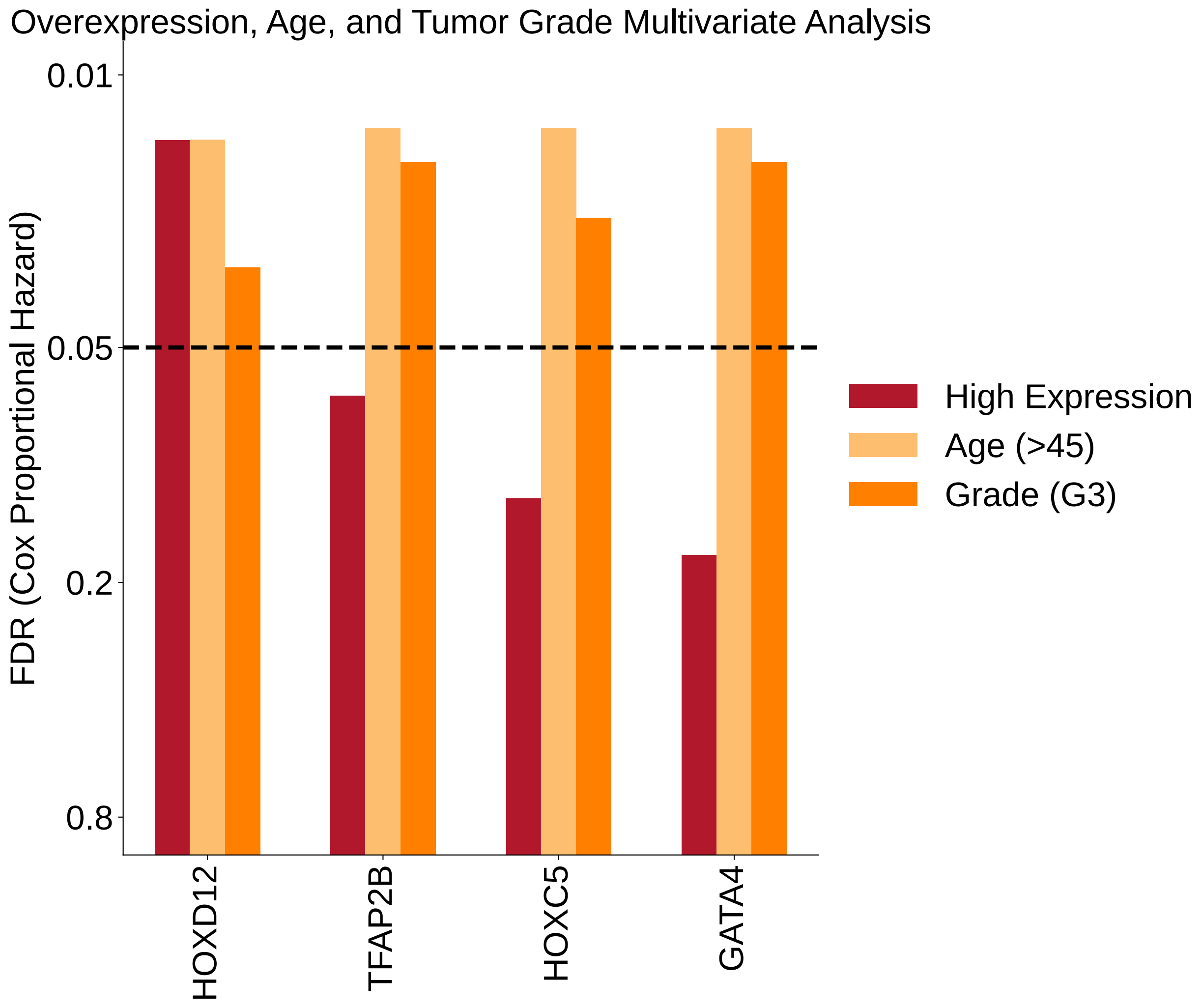

SUPPLEMENTAL FIGURE 7. *HOXD12* is the only age-associated gene whose elevated expression is independently prognostic of oligodendroglioma age and tumor WHO grade. Of the four age-associated genes whose elevated expression (greater than median) is significantly prognostic in TCGA oligodendroglioma univariate tests, *HOXD12* is the only gene whose elevated expression is significantly prognostic independent of age and WHO grade after p-value correction (FDR)**.**

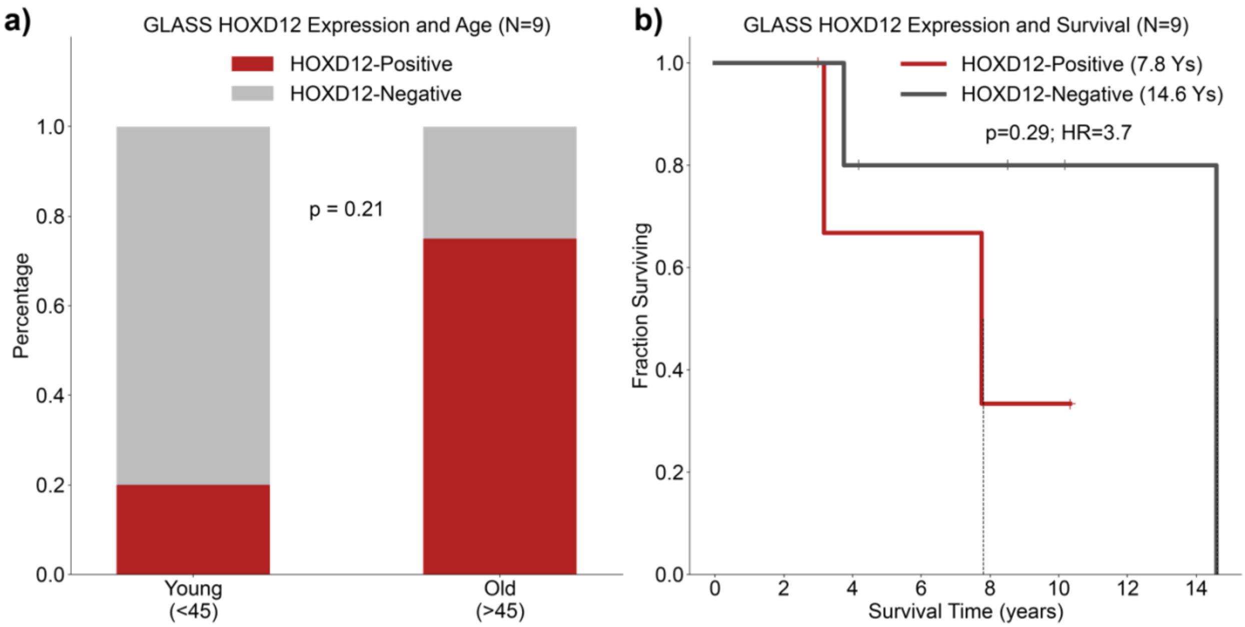

SUPPLEMENTAL FIGURE 8. ***HOXD12-positive expression status shows trends toward age and survival association in the GLASS dataset*.** **a)** Even though there are only 9 GLASS oligodendroglioma patients with RNA-seq data, 3 of the 4 older patients harbor HOXD12-positive expression status while only 1 of 5 younger patients harbor HOXD12-positive expression status. **b)** Similarly, despite the small size of the GLASS dataset, patients with HOXD12-positive expression status trend toward worse survival.

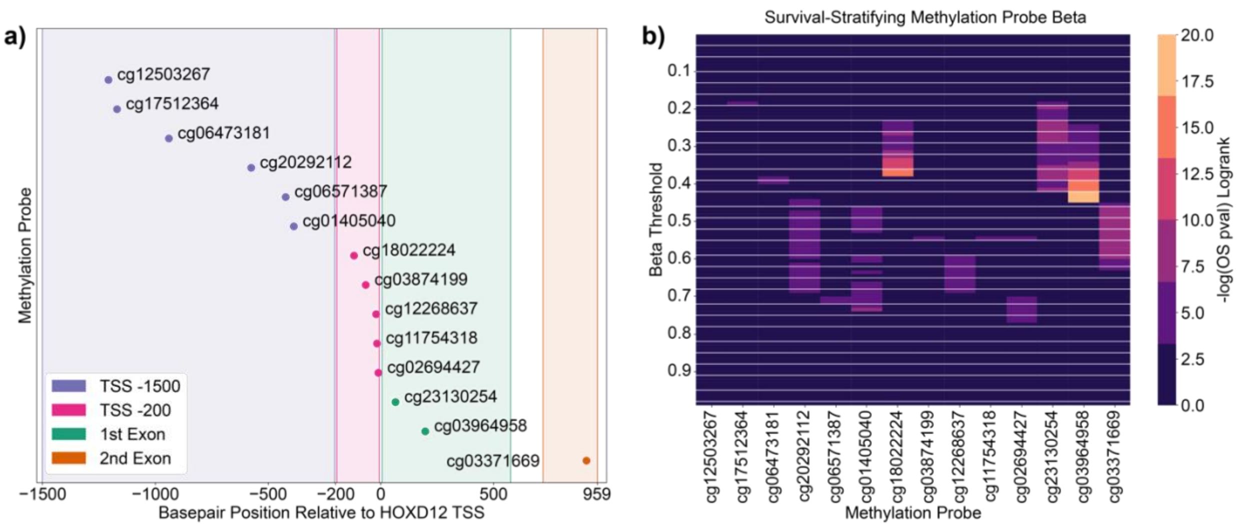

SUPPLEMENTAL FIGURE 9. ***Selecting thresholds for HOXD12-associated DNA methylation probes for significance testing*****.** **a)** *HOXD12*-associated methylation probes were classified according to their genomic position relative to *HOXD12's* transcription start site (TSS). **b)** Beta value thresholds that best-stratified survival in the TCGA for each HOXD12-associated probe was chosen for later inter-probe comparisons.

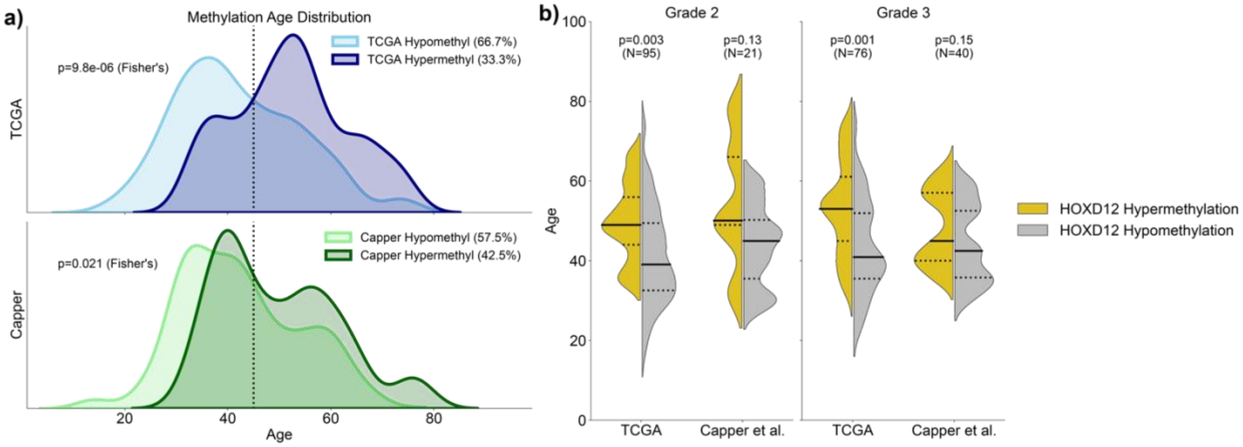

SUPPLEMENTAL FIGURE 10. a) *HOXD12* gene body hypermethylation was associated with age in the TCGA and Capper et al. cohorts (Fisher's). **b)** *HOXD12* gene body hypermethylation was associated with age in the TCGA and Capper et al. cohorts in both WHO grade 2 and WHO grade 3 tumors (Mann-Whitney U).

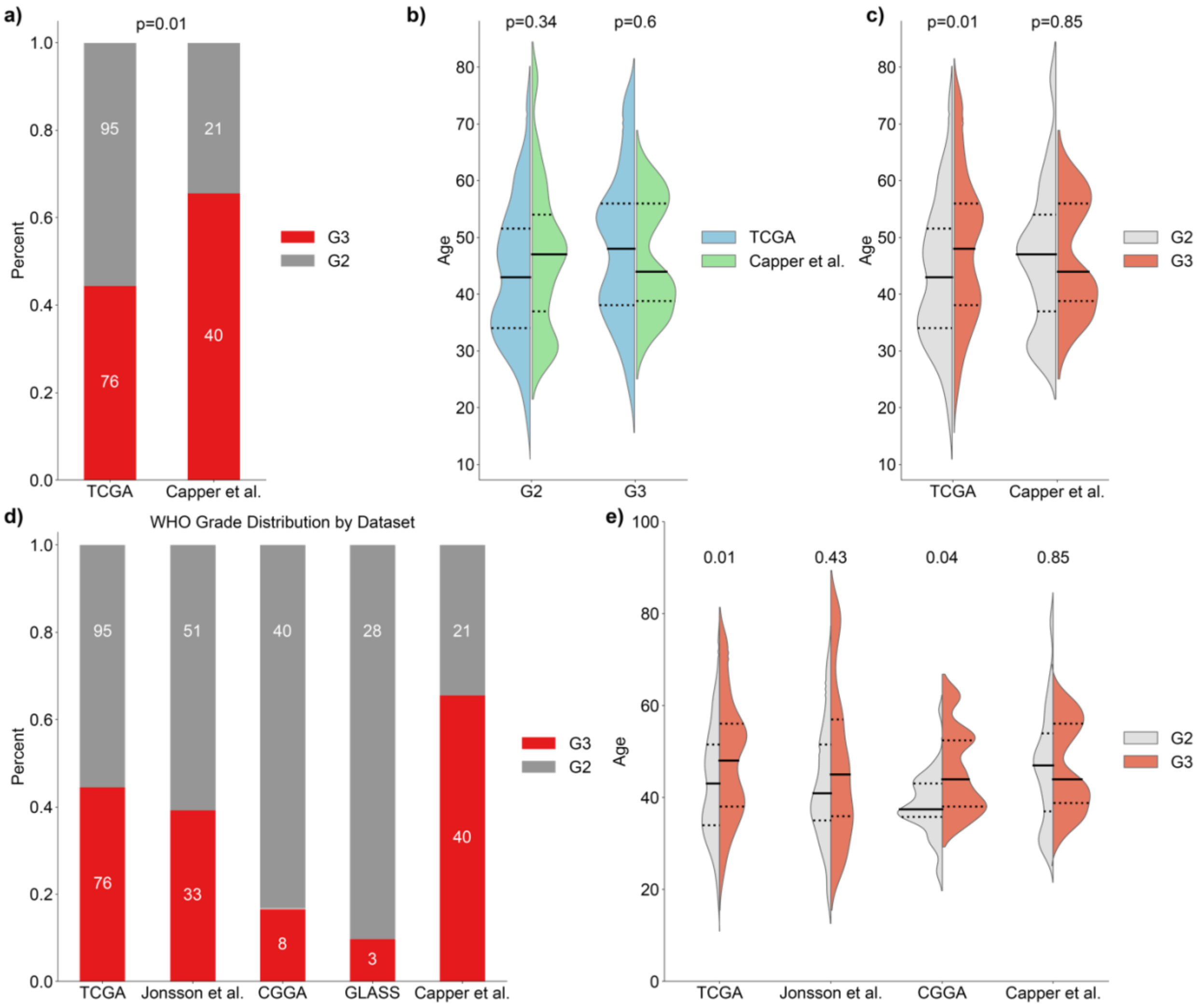

SUPPLEMENTAL FIGURE 11. The Capper et al. cohort was enriched for younger, higher-WHO grade tumors compared to all other datasets we analyzed, including the TCGA. **a)** The Capper et al. dataset had disproportionally higher-grade tumors than the TCGA (Fisher's). **b)** Capper et al. WHO grade 2 oligodendrogliomas were older than TCGA g WHO rade 2 oligodendrogliomas, and Capper et al. WHO grade 3 oligodendrogliomas were younger than TCGA WHO grade 3 oligodendroglioma (Mann-Whitney U). **c)** Unlike the TCGA, the median age of WHO grade 2 Capper et al. oligodendrogliomas was higher than the median age of WHO grade 3 Capper et al. oligodendrogliomas (Mann-Whitney U). **d)** Unlike any other dataset we have analyzed, the majority of Capper et al. oligodendrogliomas were WHO grade 3. **e)** Unlike any other dataset with more than five WHO grade 3 oligodendrogliomas we analyzed, WHO grade 3 oligodendrogliomas were younger than WHO grade 2 oligodendrogliomas in the Capper et al. cohort (Mann-Whitney U).

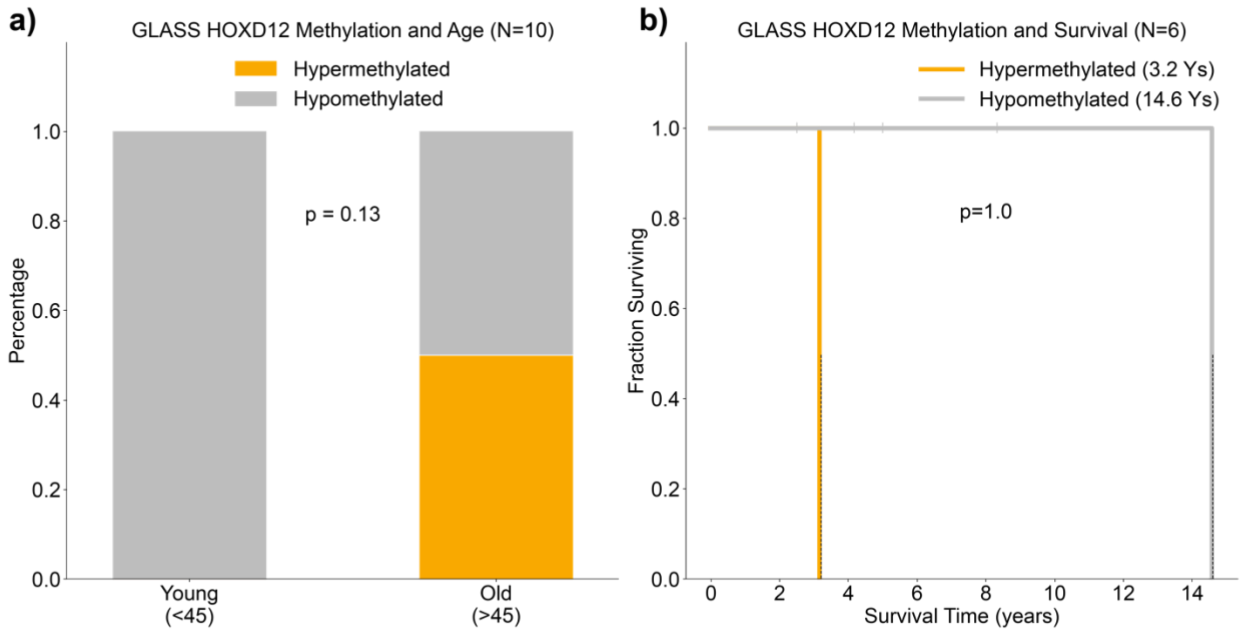

SUPPLEMENTAL FIGURE 12. ***HOXD12 gene-body hypermethylation points toward age and survival association in the GLASS dataset*.** **a)** Even though there are only 10 GLASS oligodendroglioma patients with primary tumor DNA methylation data, all HOXD12 hypermethylated patients (N=2) are older. **b)** Similarly, despite the small size of the GLASS dataset, HOXD12-positive expression status points toward worse survival.

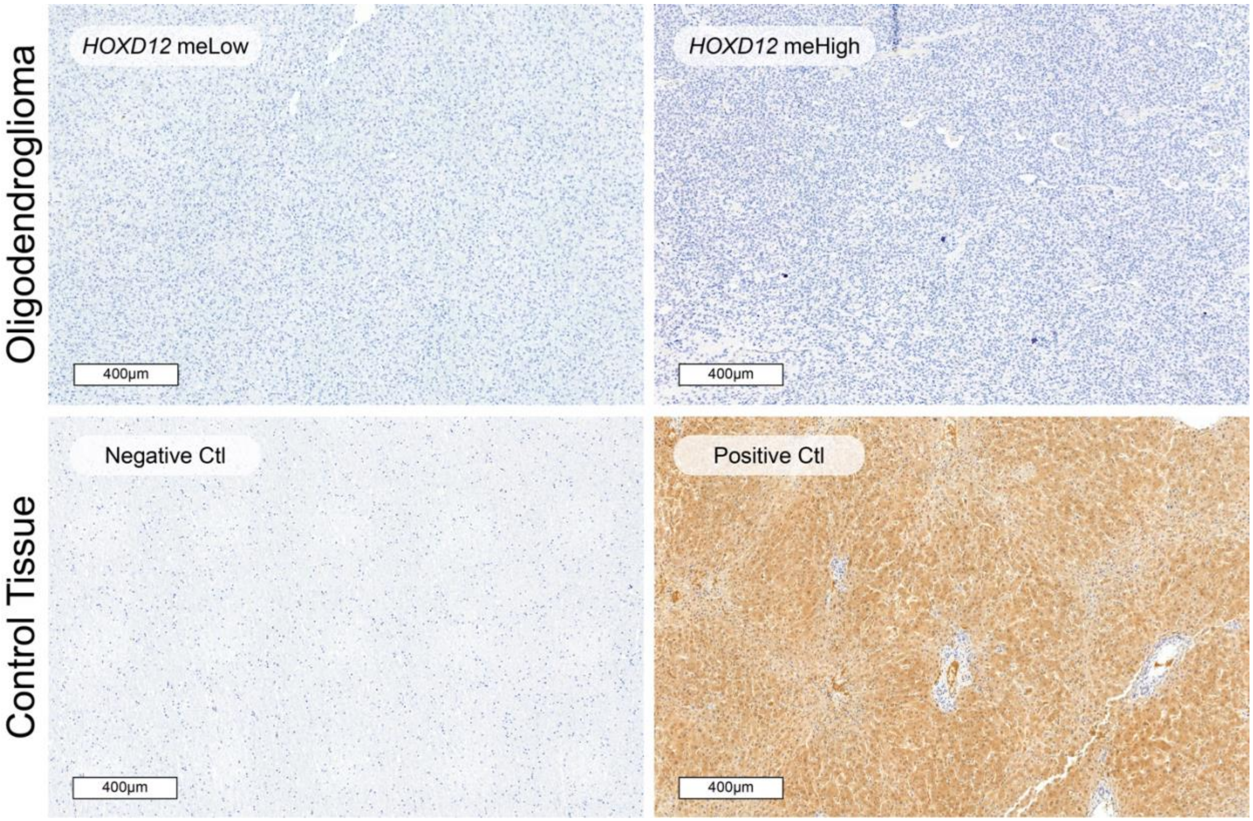

SUPPLEMENTAL FIGURE 13. *HOXD12* immunohistochemistry is not a sensitive surrogate marker for *HOXD12* methylation status. There is lack of detectable *HOXD12* immunostaining in oligodendrogliomas with either relatively hypomethylated (meLow) or hypermethylated (meHigh) *HOXD12*, similar to normal brain (negative control tissue). *HOXD12* is reliably detected in hepatocytes (positive control) with internal negative control (vessels).

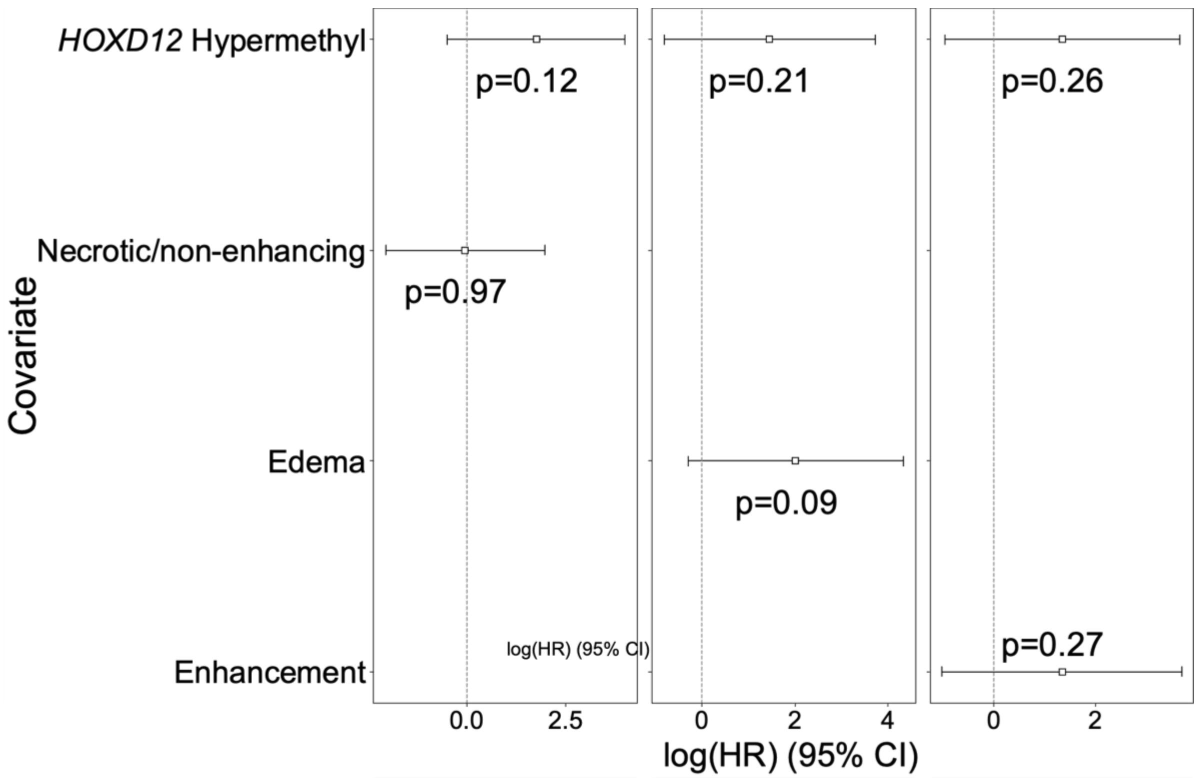

SUPPLEMENTAL FIGURE 14. *HOXD12* gene-body hypermethylation was not independently prognostic of radiographic features. Multivariate analyses showed that *HOXD12* gene-body hypermethylation was not independently prognostic of tumor necrotic/non-enhancing, edema, or enhancement volume. Whole tumor volume was omitted due to collinearity.

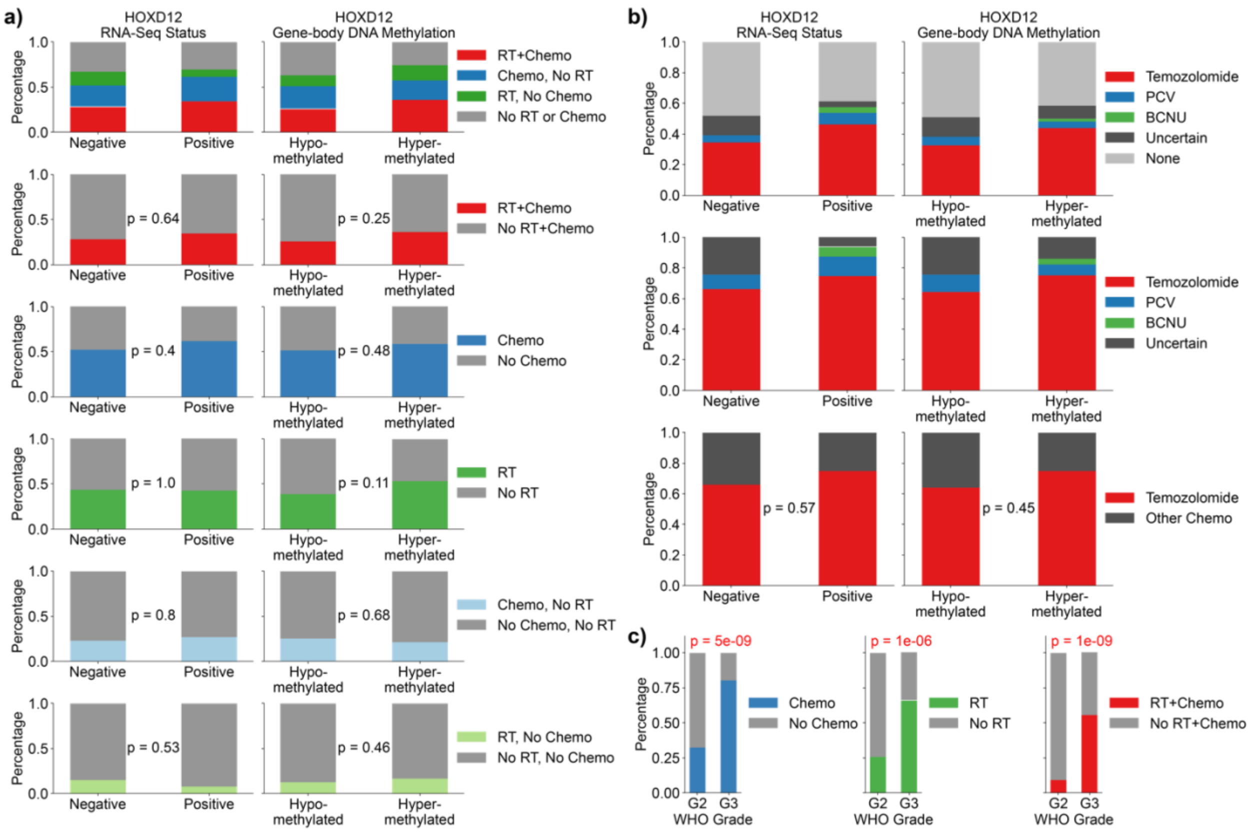

SUPPLEMENTAL FIGURE 15. No significant treatment differences were observed between oligodendroglioma patients with or without *HOXD12* gene-body hypermethylation or with or without HOXD12-positive expression status in the TCGA. **a)** No significant differences in the administration of adjuvant chemoradiation, adjuvant chemotherapy, adjuvant radiation, adjuvant chemotherapy without radiation therapy, or adjuvant radiation therapy without chemotherapy were observed between patients with or without *HOXD12* gene-body hypermethylation or between patients with or without HOXD12-positive expression status. **b)** Temozolomide was the dominant chemotherapy agent and was not significantly more commonly administered in patients with or without *HOXD12* gene-body hypermethylation or between patients with or without HOXD12-positive expression status**. c)** WHO grade 2 TCGA oligodendrogliomas were treated with chemoradiation, chemotherapy, and radiation dramatically less frequently than WHO grade 3 TCGA oligodendrogliomas.

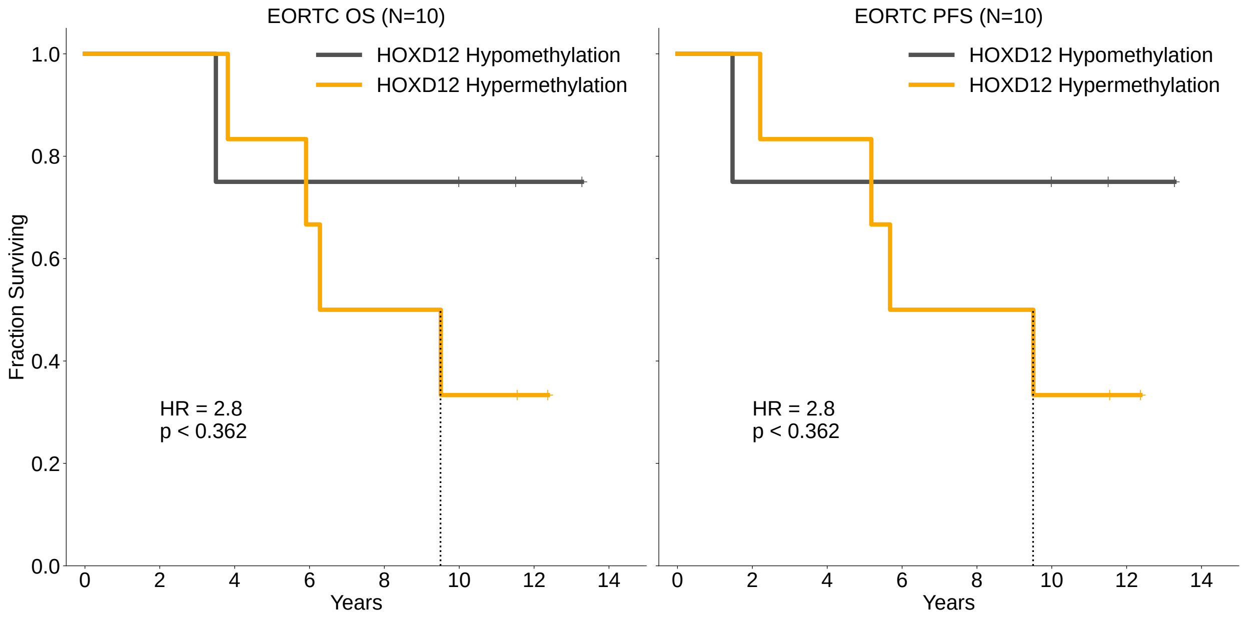

SUPPLEMENTAL FIGURE 16. HOXD12 gene body hypermethylation in EORTC 26951 Phase III 1p/19q-codeleted oligodendrogliomas treated with PCV. A weak trend toward worse survival among *HOXD12* gene body hypermethylated patients was observed in both overall and progression-free survival.

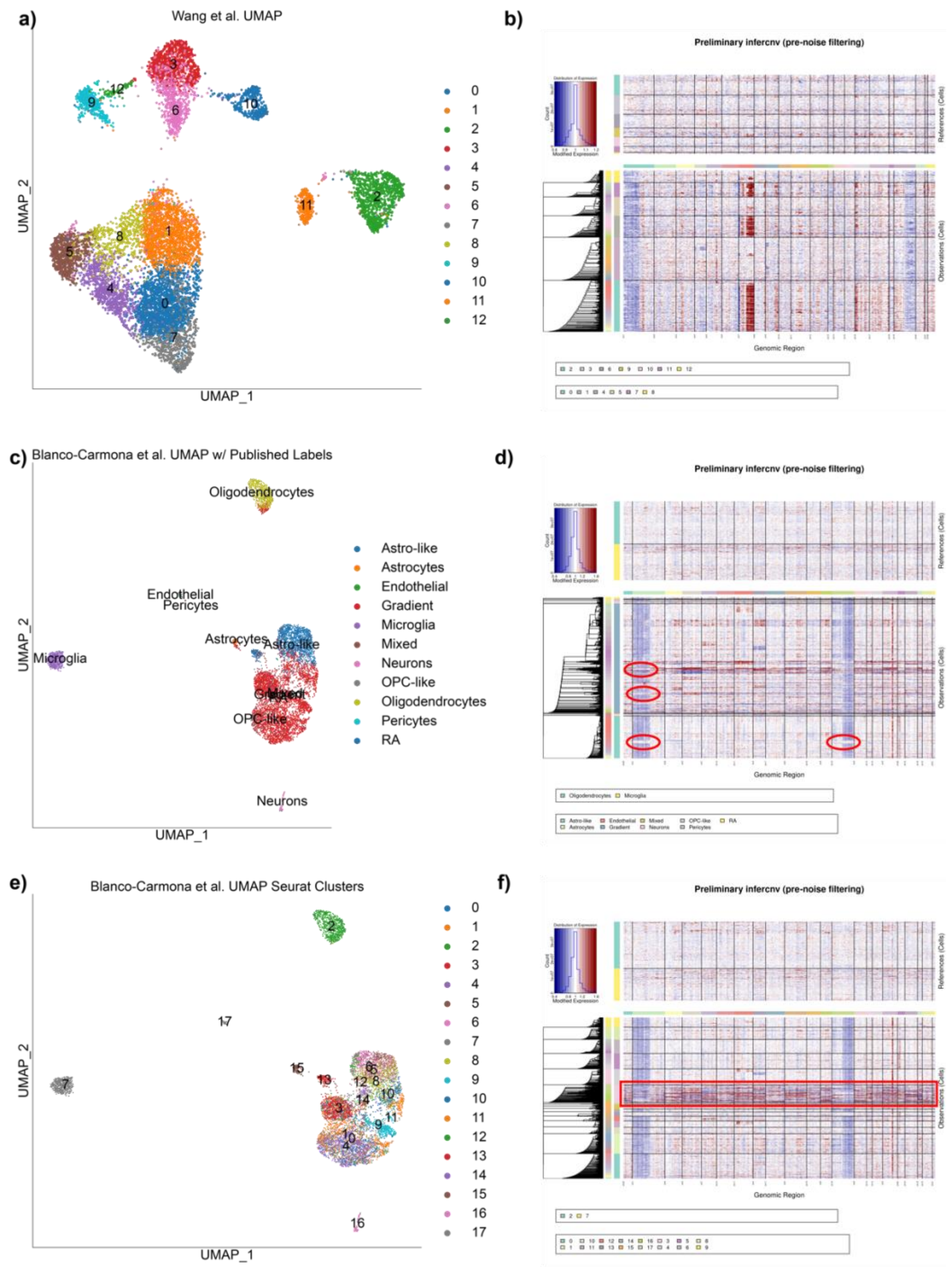

SUPPLEMENTAL FIGURE 17. ***Nuclei from snRNA-seq were determined to be neoplastic or non-neoplastic based on the presence or absence of a 1p/19q co-deletion, as computed by inferCNV*****.** **a)** UMAP embedding of estimated snRNA-seq data published by Wang et al. annotated by Seurat generated clusters. **b)** Estimated somatic copy number alterations (SCNA) calls from estimated snRNA-seq data published by Wang et al. grouped by cluster labels shown on the UMAP

embedding in (a). **c)** UMAP embedding of snRNA-seq published by Blanco Carmona et al. colored by non-neoplastic cell types and neoplastic cell states annotations published in the same study. **d)** Estimated SCNA calls from snRNA-seq data published by Blanco Carmona et al. grouped by cell type/state annotations published by Blanco Carmona et al. shown (c). Circled in red are labeled neoplastic cells that lack a 1p/19q codeletion and are likely mislabeled; for this reason, we reclassified nuclei as non-neoplastic or neoplastic. **e)** UMAP embedding of snRNA-seq published by Blanco Carmona et al. colored by Seurat generated clusters. **f)** Estimated SCNA calls from snRNA-seq data published by Blanco Carmona et al. grouped by cluster labels shown on the UMAP embedding in (e).

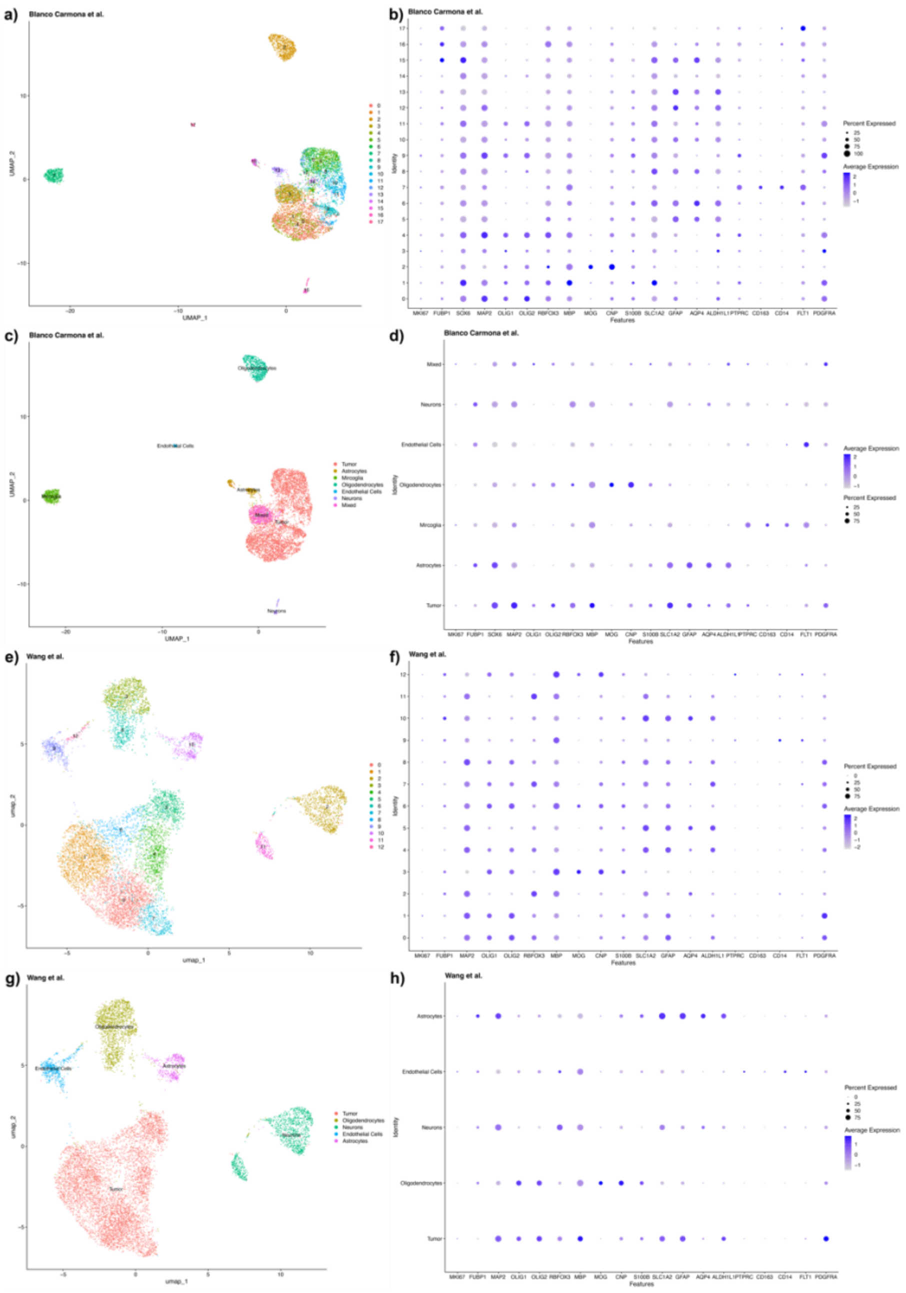

SUPPLEMENTAL FIGURE 18. ***Cell type determination by cell type markers*****. a)** Blanco-Carmona et al. UMAP embedding of snRNA-seq data labeled by Seurat-generated clusters. **b)** Dot plot of cell type markers by clusters made from Blanco-Carmona et al. snRNA-seq data. **c)** Blanco-Carmona et al. UMAP embedding of snRNA-seq data labeled cell type marker-deduced cell types. **d)** Dot plot of cell type markers by deduced cell type made from Blanco-Carmona et al. snRNA-seq data. **e)** Wang et al. UMAP embedding of estimated snRNA-seq data labeled by Seurat-generated clusters. **f)** Dot plot of cell type markers by clusters made from Wang et al. estimated snRNA-seq data. **g)** Wang et al. UMAP embedding of estimated snRNA-seq data

labeled cell marker-deduced cell types. **h)** Dot plot of cell type markers by deduced cell type made from Wang et al. estimated snRNA-seq data.

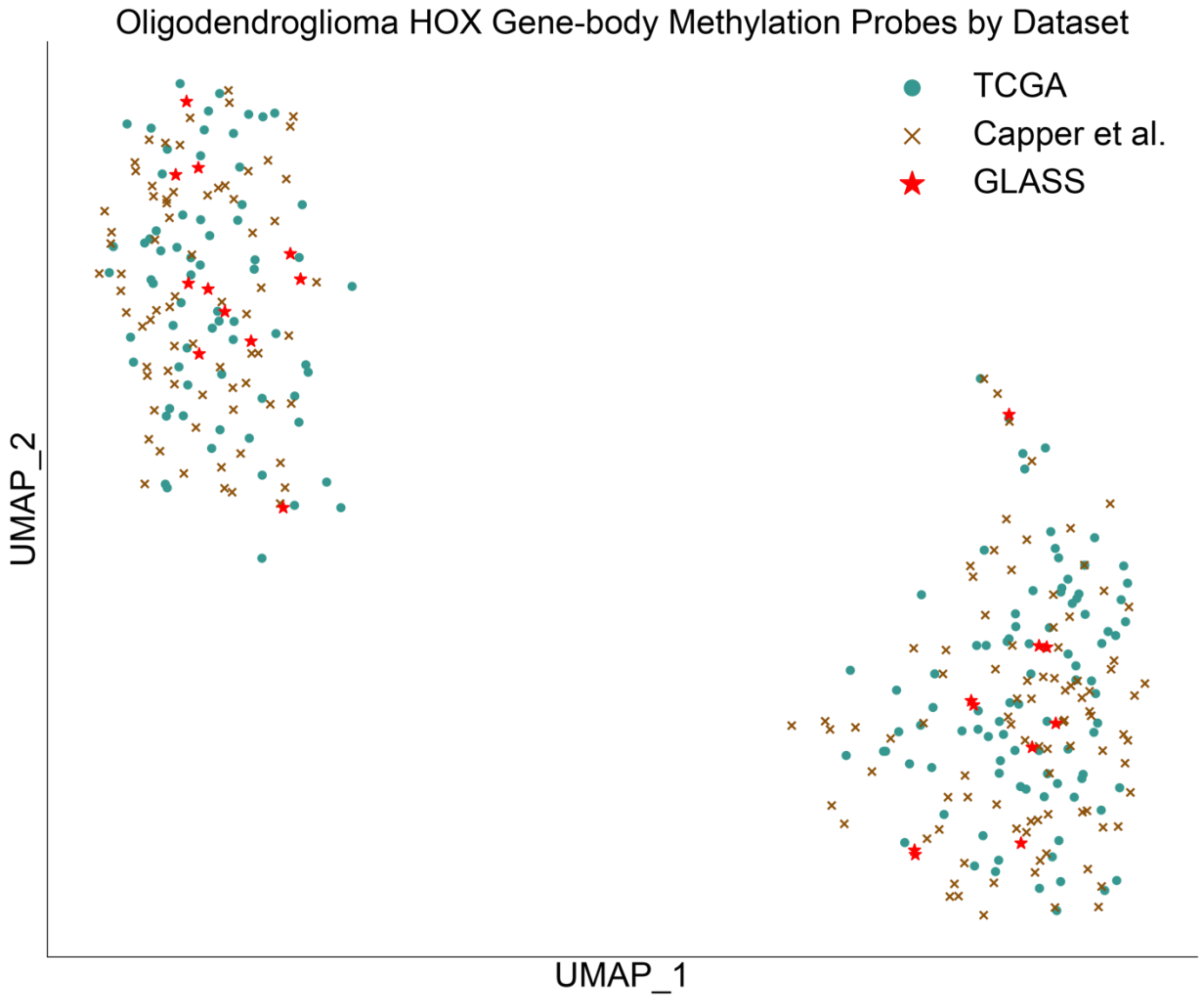

SUPPLEMENTAL FIGURE 19. ***Pan-HOX UMAP colored in by dataset***. Datasets appear to mix well together on a UMAP embedding of HOX gene-body DNA methylation data.

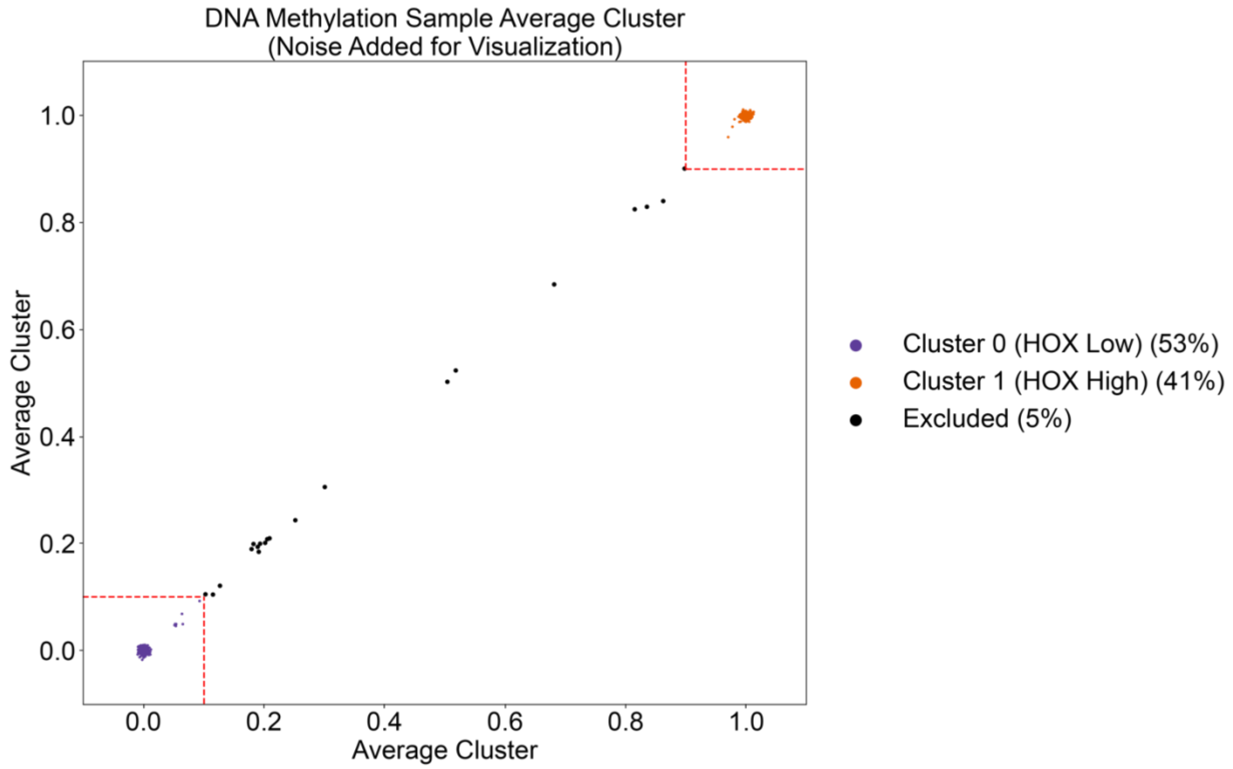

SUPPLEMENTAL FIGURE 20. ***Quality control for DNA methylation samples in UMAP embedding-based clusters***. UMAP models were trained and evaluated 1000 times with different random initializations. Clustering of each resulting embedding gave two clusters, one with low mean HOX gene-body DNA methylation (Cluster 0) and one with high HOX gene-body DNA methylation (Cluster 1). For each sample, the average cluster was calculated by taking the mean cluster membership over all 1000 UMAP embeddings; this resulted in a number between 0 and 1 for each sample. We selected samples that belonged to cluster 0 (HOX Low) in at least 90% of UMAPs and samples that belonged to cluster 1 (HOX High) in at least 90% of UMAPs. In total, the 5% of our data that did not meet these conditions was excluded.

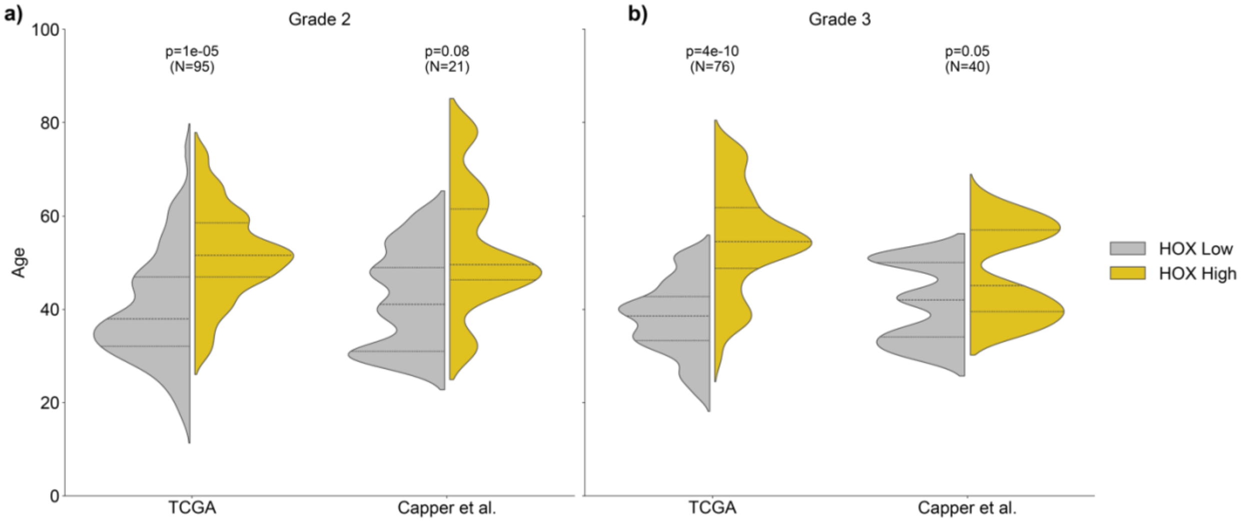

SUPPLEMENTAL FIGURE 21. *HOX* gene body DNA methylation clusters (HOX-high and HOX-low) were associated with age in the TCGA and the Capper et al. cohort across WHO grades. a,b) HOX-high status was associated with age in the TCGA in WHO grade 2 and WHO grade 3 oligodendrogliomas, and Capper et al. tumors displayed the same trends (Mann-Whitney U).

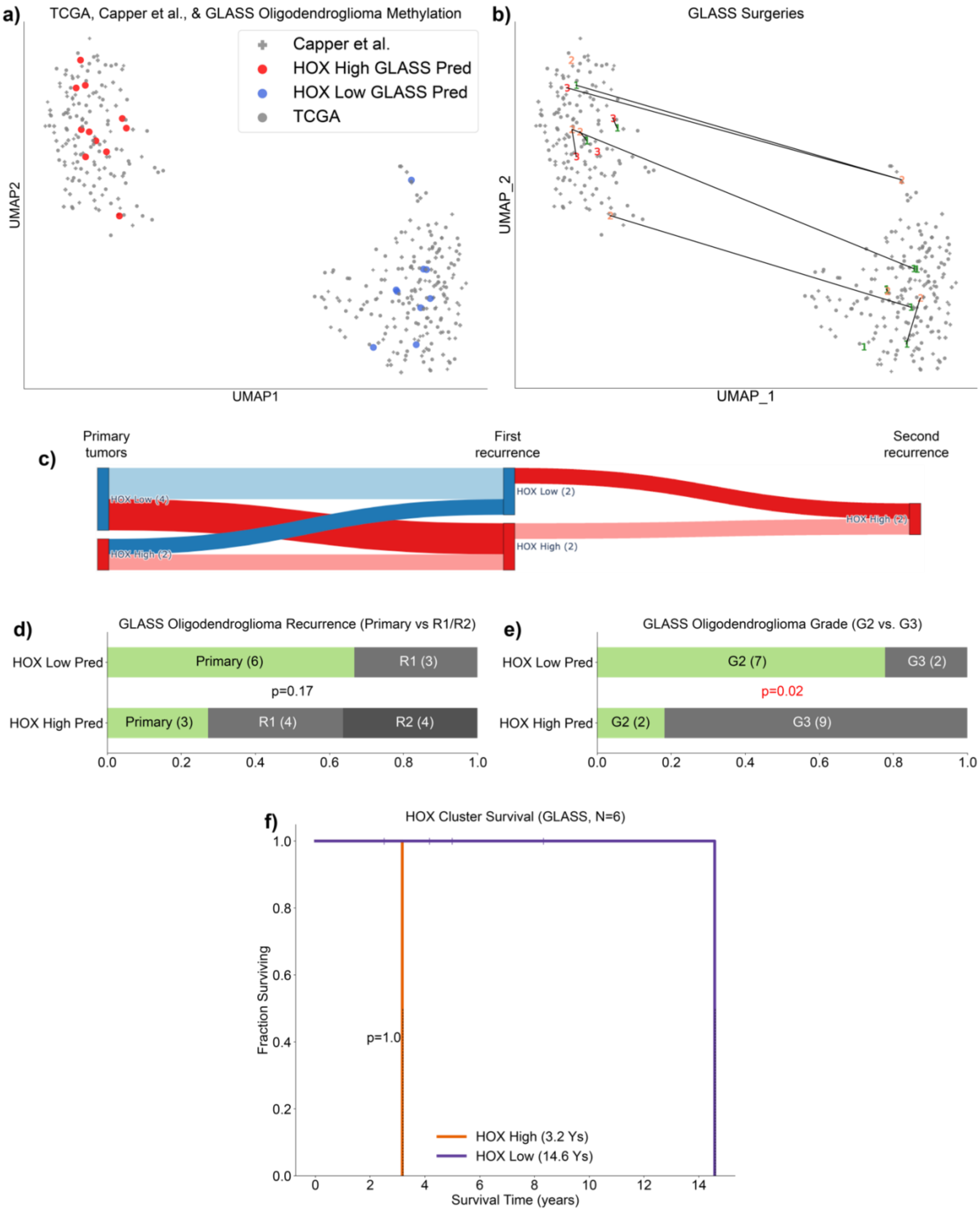

SUPPLEMENTAL FIGURE 22. ***GLASS tumors tended to migrate from the HOX-low cluster to the HOX-high cluster during evolution*****. a)** UMAP embedding of batch-corrected HOX gene-body DNA methylation for TCGA, Capper et al., and GLASS oligodendroglioma samples with GLASS samples highlighted in the HOX high (red) cluster and HOX low (blue) cluster. **b)** Lines

connecting GLASS samples from the same patient are shown on the UMAP embedding described in (a). **c)** A snaky plot shows that most GLASS primary tumors begin in the HOX low cluster and tend to move to the HOX high tumor as they progress. **d)** GLASS samples in the HOX high cluster tend to be recurrent (R1 or R2) vs. GLASS samples in the HOX low cluster, which tend to be primary tumors (TP). **e)** GLASS samples in the HOX high cluster have higher WHO grade (G3) compared to GLASS samples in the HOX low cluster. **f)** Although the sample size is too small to draw conclusions, GLASS patients in the HOX-high cluster had shorter overall survival compared to GLASS patients in the HOX-low cluster.

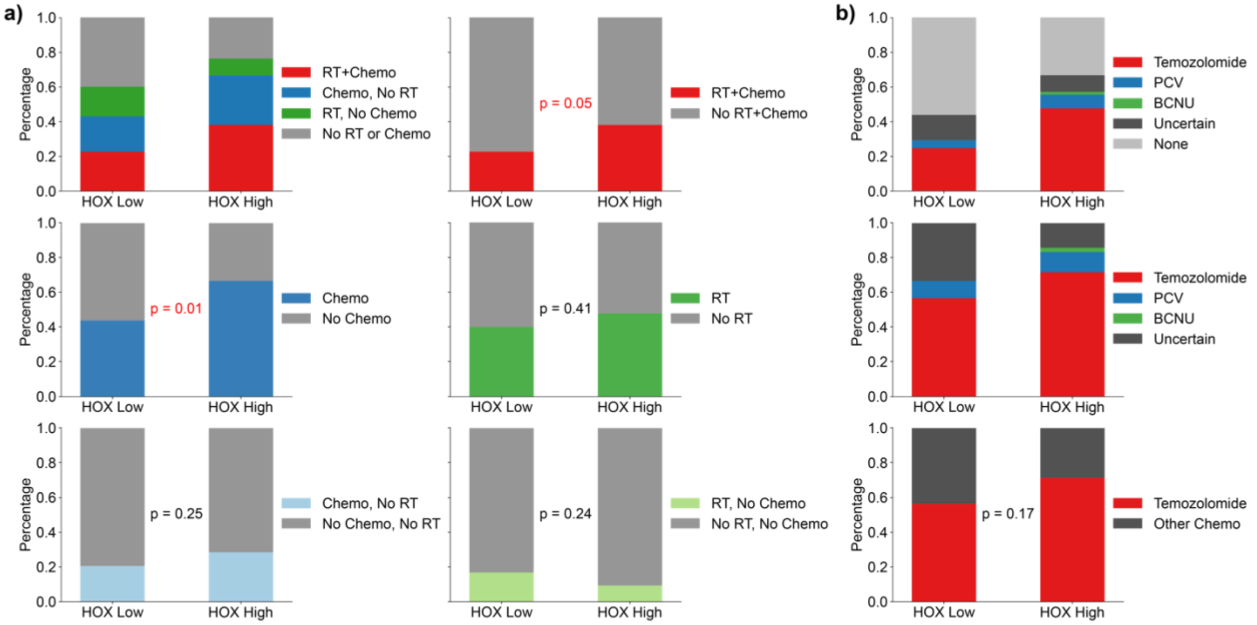

SUPPLEMENTAL FIGURE 23. Treatment differences are unlikely to explain the survival differences between HOX-high and HOX-low oligodendroglioma. a) The only significant difference in treatment between HOX-high patients and HOX-low patients was the administration of adjuvant chemoradiation and adjuvant chemotherapy. In both cases, the more aggressive HOX-high patients more commonly received therapy, which is the opposite relationship is necessary for treatment differences to explain why HOX-high patients had worse outcomes than HOX-low tumors. **b)** Temozolomide was the dominant chemotherapy agent and was not significantly more commonly administered in patients with or without HOX-high or HOX-low status.

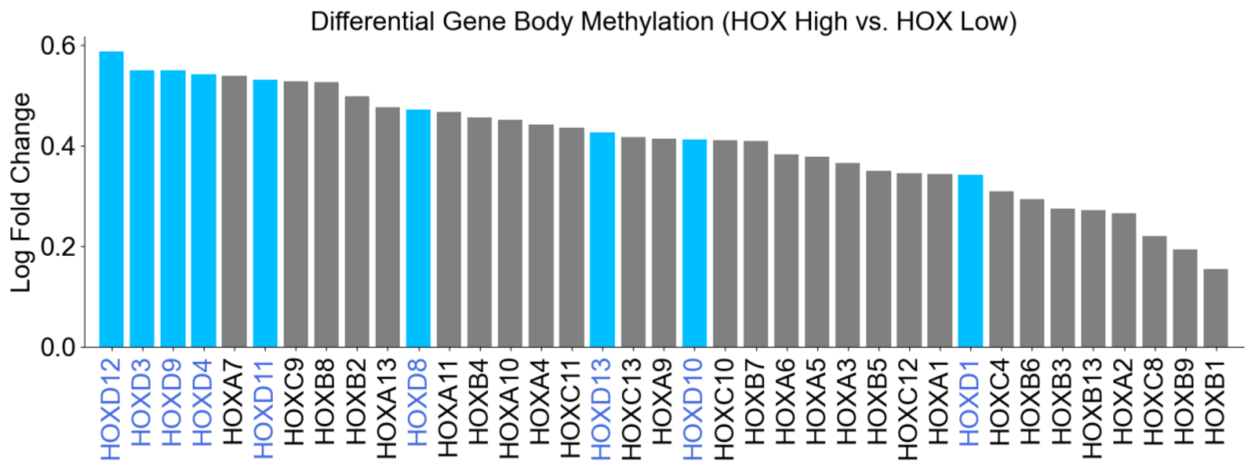

SUPPLEMENTAL FIGURE 24. ***HOXD12 was the most differentially gene-body methylated HOX gene between the HOX-high and HOX-low pan-HOX gene-body DNA methylation clusters.*** In a differential methylation analysis between HOX gene-body DNA methylation clusters, *HOXD12* (followed by several other genes on the HOXD locus) had the highest log2 fold change between of any HOX gene, all with higher DNA methylation values in the HOX-high cluster.
